# Epithelial cells derived exosomal miR-203a-3p facilitates stromal inflammation of type IIIA chronic prostatitis/chronic pelvic pain syndrome by targeting DUSP5 and increasing MCP-1 generation

**DOI:** 10.1186/s12951-024-02513-5

**Published:** 2024-05-10

**Authors:** Guojing Song, Fuhan Zhao, Rongrong Ni, Bingqian Deng, Saipeng Chen, Ruimin Hu, Jun Zheng, Yiji Peng, Heting Liu, Yang Luo, Zhansong Zhou, Gang Huang, Wenhao Shen

**Affiliations:** 1 Department of Urology, Southwest Hospital, Army Medical University (Third Military Medical University), Chongqing, 400038 China; 2 Department of Biochemistry and Molecular Biology, College of Basic Medical Science, Army Medical University (Third Military Medical University), Chongqing, 400038 China; 3 Department of Center of Smart Laboratory and Molecular Medicine, School of Medicine, Chongqing University, Chongqing, 400044 China

**Keywords:** CP/CPPS-A, Exosomes, miR-203a-3p, DUSP5, Inflammation

## Abstract

Increased proinflammatory cytokines and infiltration of inflammatory cells in the stroma are important pathological features of type IIIA chronic prostatitis/chronic pelvic pain syndrome (CP/CPPS-A), and the interaction between stromal cells and other cells in the inflammatory microenvironment is closely related to the inflammatory process of CP/CPPS-A. However, the interaction between stromal and epithelial cells remains unclear. In this study, inflammatory prostate epithelial cells (PECs) released miR-203a-3p-rich exosomes and facilitated prostate stromal cells (PSCs) inflammation by upregulating MCP-1 expression. Mechanistically, DUSP5 was identified as a novel target gene of miR-203a-3p and regulated PSCs inflammation through the ERK1/2/MCP-1 signaling pathway. Meanwhile, the effect of exosomes derived from prostatic fluids of CP/CPPS-A patients was consistent with that of exosomes derived from inflammatory PECs. Importantly, we demonstrated that miR-203a-3p antagomirs-loaded exosomes derived from PECs targeted the prostate and alleviated prostatitis by inhibiting the DUSP5-ERK1/2 pathway. Collectively, our findings provide new insights into underlying the interaction between PECs and PSCs in CP/CPPS-A, providing a promising therapeutic strategy for CP/CPPS-A.

**Supplementary Information:**

The online version contains supplementary material available at 10.1186/s12951-024-02513-5.

## Introduction

Chronic prostatitis/chronic pelvic pain syndrome (CP/CPPS) is a widespread condition that affects men across a broad age spectrum, characterized by significant symptoms such as pelvic pain, varying degrees of voiding issues, and sexual dysfunction [1, 2]. However, the underlying causes, pathogenesis, and the most effective treatment for CP/CPPS remain poorly understood [3, 4]. While the exact pathophysiology mechanism of CP/CPPS is not well known, it has been demonstrated that inflammatory factors secreted by stromal cells play a critical role [5, 6]. Some scholars even believe that CP/CPPS could be defined as a prostatic stromal disease [7]. Previous studies have confirmed that the interaction between stromal cells and other cells in the inflammatory microenvironment is closely linked to the inflammatory process of CP/CPPS [8, 9]. CP/CPPS is further categorized into IIIA and IIIB, with Type IIIA referring to inflammatory CP/CPPS. Patients with Type IIIA CP/CPPS exhibit more severe and frequent symptoms compared to those with noninflammatory CP/CPPS (Type IIIB) [10]. Nevertheless, the interaction between prostate stromal cells (PSCs) and prostate epithelial cells (PECs), which are the primary cells responsible for secreting prostate fluid and maintaining prostate homeostasis, remains unclear in the context of CP/CPPS-A [11, 12]. Therefore, elucidating the interaction between PECs and PSCs in the inflammatory microenvironment is crucial to improving the understanding and treatment of CP/CPPS-A.

Exosomes are a subtype of extracellular vesicles, typically ranging in diameter from 40 to 160 nm, actively released by various cells into extracellular fluids, such as blood, cerebrospinal fluid, saliva, urine, and prostatic fluid [13–15]. Exosomes play a crucial role in mediating intercellular communication by transporting specific proteins, lipids, microRNAs (miRNAs), and genetic materials from their parent cells to recipient cells [16, 17]. In particular, the role of exosomal non-coding RNAs in disease has garnered extensive attention, with growing evidence indicating that exosomal miRNAs are closely involved in regulating disease progression [18]. For instance, during the initial phase of vascular inflammation, endothelial extracellular vesicles that respond early play a role in promoting monocyte activation by transferring miR-126-5p and miR-212-3p [19]. Similarly, highly metastatic colorectal cancer cells-derived extracellular vesicles rich in miR-181a-5p could activate hepatic stellate cells, thereby facilitating liver metastasis in colorectal cancer [20]. However, it remains unclear whether epithelial cells can influence stromal cells via exosomal miRNAs in CP/CPPS-A.

In this study, we found for the first time that prostatic fluid of CP/CPPS-A patients and lipopolysaccharide (LPS)-stimulated PECs contained miR-203a-3p-rich exosomes, which were absorbed by stromal cells and subsequently promoted inflammation. Mechanistically, we identified that DUSP5 was a novel target gene of miR-203a-3p. The downregulation of DUSP5 by miR-203a-3p increased the phosphorylation of ERK1/2 and subsequently induced MCP-1 expression, thus promoting stromal cell inflammation. Finally, we developed miR-203a-3p antagomirs-loaded exosomes derived from PECs, which could target the prostate and alleviate prostatitis by inhibiting the DUSP5-ERK1/2-MCP-1 pathway. Our results suggest that inflammatory PEC-derived exosomes promote stromal cell inflammation via the miR-203a-3p/DUSP5/MCP-1 axis, providing a promising strategy for targeted treatment of CP/CPPS-A.

## Materials and methods

### Collection of human prostatic fluid samples

This study involved twenty prostatic fluid samples from CP/CPPS-A patients diagnosed at the Department of Urology, Southwest Hospital, Army Medical University (Chongqing, China). Additionally, twenty healthy volunteers were randomly recruited from the general population for this study. The inclusion and exclusion criteria for patients with CP/CPPS-A and healthy adult males were implemented as previously reported [21]. Written informed consent was obtained from each participant. The study protocol adhered to the ethical guidelines of the 1975 Declaration of Helsinki and was approved by the Ethics Committee of Southwest Hospital of Army Medical University (Ethics approval number: KY201801).

### Cell culture and collection of cell supernatant

The human prostate epithelial cell line (PEC: RWPE-1 cell) and human prostate stromal cell line (PSC: WPMY-1 cell) were purchased from the American Type Culture Collection (ATCC, USA) and tested for no mycoplasma contamination. WPMY-1 cells were cultured in DMEM medium (Gibco, USA), supplemented with 10% (v/v) fetal bovine serum (FBS) (Gibco, USA). RWPE-1 cells were cultured in keratinocyte growth medium supplemented with human recombinant epidermal growth factor (5 ng/mL) and bovine pituitary extract (0.05 mg/mL) (ScienCell, USA). Cells were maintained at 37 °C in a 5% CO_2_ environment. To collect cell supernatant, RWPE-1 cells were grown to 70% confluence in five 175 cm^2^ flasks (4–8 × 10^7^ cells). The growth medium was then replaced with an equivalent medium supplemented with exosomes-depleted FBS (obtained by ultracentrifuging FBS supernatant at 100,000×g for 16 h) with corresponding treatment (100 ng/mL LPS, 100 nM miRNA mimics, 100 nM miRNA inhibitors or negative controls). LPS was purchased from Sigma (USA). miR-203a-3p mimics, miR-203a-3p inhibitors, and negative controls were designed and synthesized by Genepharma (China), with target sequences listed in Table S1. After 24 h, the conditioned medium was harvested.

### Isolation and characterization of exosomes

Exosomes were isolated by using differential ultracentrifugation [22]. Briefly, the prostatic fluid sample or cell supernatant was first centrifuged at 300×g for 10 min to pellet floating cells, followed by centrifugation at 2,000×g for 10 min to pellet cellular debris. The supernatant was then centrifuged at 10,000×g for 30 min to pellet apoptotic bodies and microparticles larger than 1 μm. The resulting supernatant was filtered through a 0.22 μm filter and ultracentrifuged at 100,000×g for 90 min (Beckman Coulter, USA). The resulting exosomal pellets were pooled, washed with phosphate buffer saline (PBS), and ultracentrifuged again at 100,000×g. The final exosomal pellets were resuspended in 100 µL of PBS and stored at -80 °C. Using this method, exosomes were isolated from RWPE-1 cells, LPS-stimulated RWPE-1 cells, normal prostatic fluids, and CP/CPPS-A prostatic fluids, yielding four distinct exosome types: NC-Exos, LPS-Exos, Normal-Exos, and CP/CPPS-A-Exos. For the detection of exosomal particle size, 100 µL of exosomes was diluted into a final volume of 1 mL and subjected to Nanoparticle Tracking Analysis (NTA) (Malvern, UK). The morphology of the exosomes was observed by Transmission Electron Microscope (TEM) (JEOL, Japan). Western blotting was performed to confirm the presence of exosomal biomarkers such as CD9, CD63, CD81, TSG101, Hsp70, and Calnexin (ab275018, Abcam, UK). The concentration of exosomes was determined by using an Exosome Quantitation Assay Kit (SBI, USA) following the manufacturer’s instructions.

### Animal models

The study was conducted with the approval of the Laboratory Animal Welfare and Ethics Committee of Army Medical University (Ethics approval number: AMUWEC2020939) and adhered to the US Public Health Service Policy on Humane Care and Use of Laboratory Animals. Adult male Sprague-Dawley rats, weighing 350 ± 20 g, were procured from the Experimental Animal Center of Army Medical University. The procedure entailed standard hair removal and lower abdominal skin disinfection, followed by a layered incision performed under aseptic surgical conditions to expose the bladder and the prostate situated posteriorly. A sterile micro-syringe was used to inject 20 µL of PBS, prostatic fluid derived exosomes, or RWPE-1 cells derived exosomes into the left and right ventral lobes of the prostate. Untreated rats were used as controls. After the injections, the abdominal incisions were closed in layers. Post-surgery, the rats were allowed unrestricted access to water upon regaining consciousness. One week later, the rats were euthanized, and their serum samples and prostates were collected for further analysis.

### Cell transfection

The full-length coding sequence of human DUSP5 was synthesized and cloned into the pEGFP-N1 expression vector (Tsingke, China). DUSP5 siRNAs were designed and synthesized by Genepharma (China), with target sequences listed in Table S1. For functional analysis of miR-203a-3p, the transfection was conducted using complexes of Lipofectamine 3000 (Invitrogen, USA) and miR-203a-3p inhibitors/mimics or negative controls, as per the manufacturer’s instructions. To overexpress DUSP5, WPMY-1 cells were infected with either the control pEGFP-N1 expression vector or the DUSP5-expressing pEGFP-N1 expression vector. For loss-of-function studies, WPMY-1 cells were transfected with DUSP5-specific siRNA or scrambled control oligonucleotides (si-NC).

### Detection of the uptake of exosomes

To analyze the cellular uptake of exosomes, WPMY-1 cells were seeded in a 20 mm dish (Corning, USA) at a density of 1 × 10^5^ cells/dish. Upon reaching a confluency of 30–50%, the cells were incubated with PKH67-labeled (Sigma, USA) LPS-Exos and CP/CPPS-A-Exos in DMEM medium at 37 °C for 0, 1, 2, and 4 h, respectively. The cells were then washed three times with PBS, fixed with 4% paraformaldehyde, and stained with DiR (MCE, USA) to label the cell membrane. The nuclei were stained with DAPI (Invitrogen, USA) following the manufacturer’s instructions. All images were captured using a confocal microscope (ZEISS, Germany). To analyze the effects of exosomes on cells, WPMY-1 cells were seeded in a 6-well plate at a density of 4 × 10^5^ cells/well. Once the cells reached a confluency of 70–80%, they were incubated with exosomes derived from RWPE-1 cells and prostatic fluids at a concentration of 40 µg/mL.

### miRNA expression profile chip of exosomal miRNA

Exosomes isolated from prostatic fluid samples were sent to Shanghai Genechem Company and underwent Affymetrix miRNA 4.0 microarray analysis for miRNA profiling. The significance thresholds for differentially expressed miRNA were set at adjusted *P* value < 0.01 and fold change > 2.

### Real-time quantitative PCR

Total RNA was extracted from the cells using TRIzol reagent (Invitrogen, USA). Reverse transcription of miRNAs or mRNAs was carried out using the Hairpin-it™ miRNAs RT-PCR Quantitation Kit (Genepharma, China) or PrimeScript™ RT reagent Kit (TaKaRa, Japan), respectively. The levels of miRNAs and mRNAs were then measured by using real-time quantitative PCR (qPCR), which was performed using SYBR Green dye (TaKaRa, Japan). The relative expression level of miRNAs or mRNAs was calculated by normalizing to the level of U6 or GAPDH, respectively. The relative expression level was computed using the 2^−ΔΔCT^ method. The specific primer sequences used for the qPCR reactions are listed in Table S2 and all primers were synthesized by Tsingke Biotech Company (China).

### Western blotting assay

Protein extraction was performed by using RIPA lysis buffer (Beyotime, China). The protein concentration was determined by using the BCA Protein Assay Reagent (Beyotime, China). For electrophoresis, 30 µg of protein was used. The primary antibodies used for the detection of specific proteins included the following: DUSP5 (sc-393801, Santa Cruz, USA), ERK1/2 (9102S, CST, USA), pERK1/2 (4370S, CST, USA), MCP-1 (26161-1-AP, Proteintech, China), GAPDH (60004-1-Ig, Proteintech, China). HRP-conjugated anti-mouse (SA00001-1) and anti-rabbit (SA00001-2) IgG secondary antibodies were purchased from Proteintech. The detection of the antibody-protein complexes was performed using the ECL chemiluminescence kit (Beyotime, China). The image analysis was performed using ImageJ (ImageJ 1.8, NIH, USA).

### ELISA assay

The serum and cell supernatant samples were collected from rats, mice, and cells, followed by 1,000 rpm for 20 min to isolate the supernatant. The concentration of MCP-1, IL-6, IL-15 (LiankeBio, China), CXCL1, CCL3, CCL11, IL-7, IL-17 A, IL-31 (NeoBioscience, China), and CXCL12 (JianglaiBio, China) were detected by using the corresponding ELISA kits following the manufacturer’s instructions.

### Prediction of miRNA’s target genes and analysis of KEGG signaling pathway

Several bioinformatics tools were used to predict the miRNA target genes of interest. These tools included TarBase v.8 (http://www.microrna.gr/tarbase), miRDB (https://mirdb.org/index.html), DIANA (http://diana.imis.athena-innovation.gr/DianaTools/index.php), and mirtargetlink2.0 (https://ccb-compute.cs.uni-saarland.de/mirtargetlink2/). The Kyoto Encyclopedia of Genes and Genomes (KEGG) analysis was performed to analyze miRNA pathway enrichment using DIANA-miRPath v3.0 software. The significance threshold for KEGG analysis was set at *P* < 0.05.

### Dual-luciferase reporter assay

The 3’UTR full-length cDNA of human DUSP5 was synthesized and cloned into the pmiRGLO luciferase vectors as Wild-type DUSP5 (WT), which contained the putative miR-203a-3p target binding sequence, and one of two mutated versions (MUT1 or MUT2) with altered bases in the binding site respectively (Tsingke, China). Transfection of these constructs into cells was performed using Lipofectamine 3000 reagent, following the manufacturer’s protocol. Cells were lysed after transfection 48 h, and luciferase activity was assessed using the Dual-Luciferase Reporter Assay System (Promega, USA), following the manufacturer’s instructions. The firefly luciferase activity was normalized to Renilla luciferase activity for each sample. The transfection experiments were performed in triplicate.

### Isolation of exosomes from mice prostates

Briefly, the prostate tissues were harvested from normal C57BL/6 mice (male, 25**–**30 g). Then, the tissues were cut into small pieces less than 0.1 cm^3^ and cultured in serum-free RPMI-1640 culture medium containing collagenase D and DNase I (Sigma, USA) for 45 min with gentle rotation [23]. After incubation, the supernatant was collected and filtered through a 70 μm filter. The resulting supernatant was collected for exosomes as before.

### Biodistribution of exosomes in vivo and ex vivo

The in vivo biodistribution study was conducted on normal C57BL/6 mice (male, 25**–**30 g). Mice were intravenously injected with 100 µL of PKH67-labeled exosomes (100 µg) derived from mouse prostate tissues, prostatic fluids from CP/CPPS-A patients, and healthy adult males, as well as RWPE-1 cells, respectively. In vivo, fluorescent signals emitted by the exosomes were detected 24 h post-injection employing the AniView100 system (Biolight, China). After imaging, all mice were sacrificed and major organs including the kidney, heart, liver, lung, spleen, reproductive system, and prostate were harvested. The ex vivo fluorescent signals within these organs were then measured to assess the distribution of the exosomes. All procedures were conducted under dark conditions.

### Preparation and characterization of exosomes loaded with miR-203a-3p antagomirs

Exosomes were electroporated with miRNAs (Cy5-miR-203a-3p antagomirs, miR-203a-3p antagomirs, or the negative controls) purchased from GenePharma, with target sequences listed in Table S1. The electroporation was performed at 400 V/125 µF in a 0.4 cm electroporation cuvette (Bio-Rad, USA). The ratio of exosomes to miRNAs was approximately 3 µg of exosomes to 1 pmol of miRNAs. After electroporation, any unloaded miRNAs that were attached to the surface of the exosomes were removed by performing another round of exosome isolation using the ultracentrifugation method. To quantify the amount of miR-203a-3p antagomirs that were successfully loaded into the exosomes, Cy5-tagged antagomirs were used. The fluorescent signal of the Cy5-tagged antagomirs was evaluated using a microplate reader from Thermo Fisher Scientific.

### Flow cytometry

Exosomes were attached to 4 μm aldehyde/sulfate latex beads (Invitrogen, USA) by mixing 30 µg exosomes with 10 µL beads for 15 min at room temperature with continuous rotation. Subsequently, the bead-exosomes suspension was diluted to 1 mL PBS and rotated at room temperature for 30 min. The reaction was stopped by adding 100 mM glycine and 2% bovine serum albumin (BSA) (Beyotime, China) in PBS, followed by rotating at room temperature for 30 min. Exosomes-bound beads were then washed once in 2% BSA and centrifuged for 1 min at 10,000 rpm. Beads were blocked with 10% BSA at room temperature for 30 min with rotation, followed by a second wash in 2% BSA and centrifugation for 1 min at 10,000 rpm. Subsequently, the beads were incubated with an Alexa-488-tagged anti-CD9 antibody (312104, Biolegend, USA) for 30 min at 4 °C with rotation. After incubation, the beads were centrifuged for 1 min at 10,000 rpm, the supernatant was discarded, and the beads were washed in 2% BSA and centrifuged for 1 min at 10,000 rpm. The percentage of positive beads (those bound to CD9-positive exosomes) was calculated relative to the total number of beads analyzed per sample (10,000 events).

### Analysis of anti-inflammatory efficacy of miR-203a-3p antagomirs loaded exosomes in vivo

To assess the anti-inflammatory effects of miR-203a-3p antagomirs loaded exosomes, a mouse model of chronic prostatitis was established. This was achieved by injecting 3% carrageenan (Sigma, USA) into the ventral lobes of the prostate gland. Subsequently, the mouse models were then randomly divided into three groups. Each group was intravenously injected with 100 µL of one of the following: sterile PBS, exosomes loaded with miR-203a-3p negative control antagomirs (Exo-antagomirs NC), or exosomes loaded with miR-203a-3p antagomirs (Exo-miR-203a-3p antagomirs). These injections were administered every 3 days, with a total of 3 times injections per mouse. Additionally, a group of mice without treatment was designated as the control group. Following the various treatment regimens, we collected both serum sample and prostate from each mouse for further analysis.

### Hematoxylin and eosin staining and immunofluorescence

Paraffin sections of rat and mouse prostate (n = 3) were used for hematoxylin and eosin (H&E) staining and immunofluorescence. Histological scoring of H&E-stained sections of prostates was assessed by pathologists who were blinded to the study. In brief, histological slides were evaluated by using a 6-point scoring system (Table S3). For immunofluorescence, MCP-1 (26161-1-AP, Proteintech, China) was used.

### Statistical analysis

All statistical analyses were performed using the software GraphPad Prism (version 8.00, GraphPad Software Inc, USA). For comparisons between two data groups, unpaired t-tests were used. For multi-group data comparisons, either one-way or two-way Analysis of Variance (ANOVA) followed by the Tukey HSD test was used. One-way ANOVA was used to compare the means of three or more independent groups, while two-way ANOVA was used when there were two independent variables. A *P* value less than 0.05 was considered statistically significant. *P < 0.05, **P < 0.01, ***P < 0.001, and ****P < 0.0001. And with *P* values greater than or equal to 0.05 (ns ≥ 0.05) indicating a lack of statistical significance.

## Results

### Characterization of exosomes derived from RWPE-1 cells and prostatic fluids

**Fig. 1:**
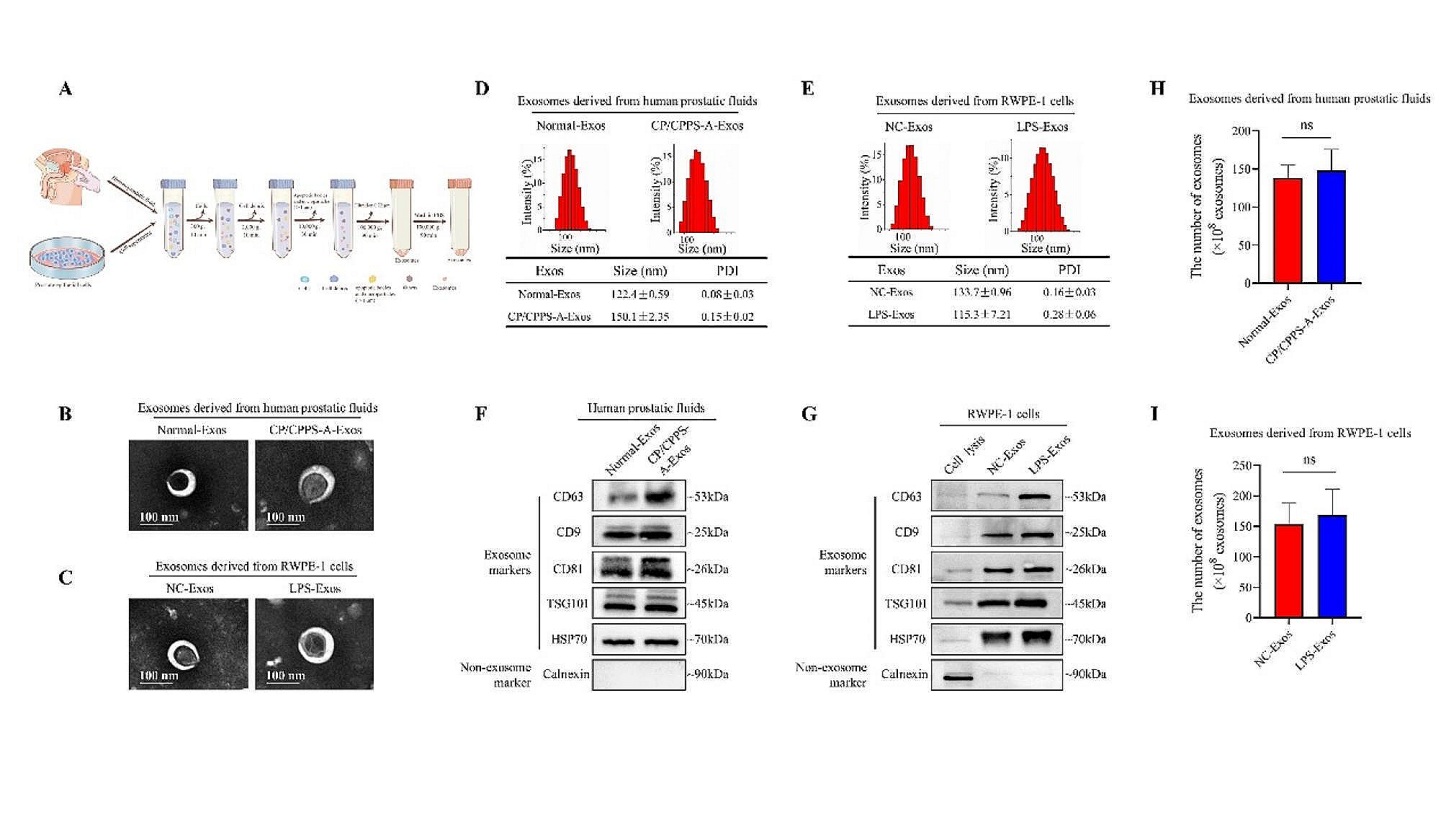
Characterization of exosomes derived from RWPE-1 cells and prostatic fluids. (A) A workflow scheme of exosomes isolated from human PECs (either basal or stimulated with 100 ng/mL LPS) and prostatic fluids (from either healthy individuals or CP/CPPS-A patients), using the ultracentrifugation method. (B)-(C) TEM analysis visualizing the morphology of the isolated exosomes, known as NC-Exos, LPS-Exos (from the supernatant of RWPE-1 cells), Normal-Exos, and CP/CPPS-A-Exos (from the prostatic fluids) (Scale bar = 100 nm). (D)-(E) NTA analysis demonstrating the particle-size distribution of the exosomes. (F)-(G) Analysis of western blotting results demonstrating protein markers CD9, CD63, CD81, HSP70, and TSG101 in exosomes derived from prostatic fluids (F) and in RWPE-1 cells lysate and exosomes derived from RWPE-1 cells (G). The endoplasmic reticulum protein, Calnexin, was used as a negative marker for exosomes. (H)-(I) Concentration of exosomes extracted from RWPE-1 cells and prostatic fluids using the ExoELISA-ULTRA Complete Kit. Statistical analyses were performed using unpaired t-tests, with *P* values greater than or equal to 0.05 (ns ≥ 0.05) indicating a lack of statistical significance

Exosomes were collected from the prostatic fluid of both healthy individuals and patients with CP/CPPS-A, as well as from the PECs (RWPE-1 cells), following a general protocol (Fig. 1A). TEM revealed that these exosomes were round-shaped extracellular vesicles surrounded by lipid bilayer membranes (Fig. 1B and C). NTA results showed that most vesicles had a median size of 130 ± 11 nm in diameter (Fig. 1D and E). Furthermore, the expression of CD9, CD63, CD81, TSG101, and HSP70 serving as typical biomarkers for exosomes was detected by western blotting, while Calnexin (an endoplasmic reticulum marker) was not detected (Fig. 1F and G). ExoELISA Quantification indicated that the number of exosomes was approximately 2 × 10^10^ per 50 µg of protein (Fig. 1H and I).

### Exosomes derived from the prostatic fluid of CP/CPPS-A patients and epithelial cells promote stromal cell inflammation

**Fig. 2:**
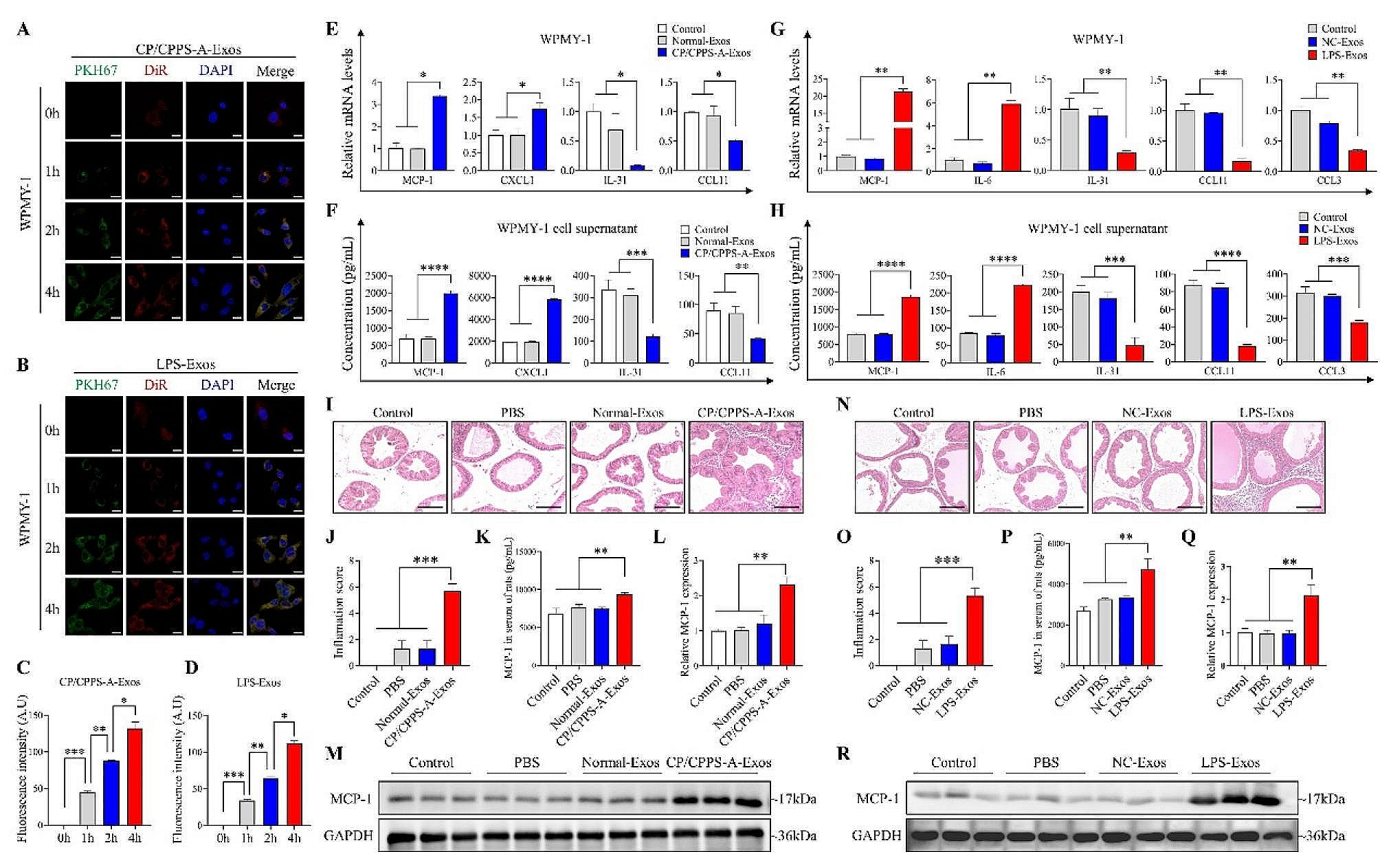
The proinflammatory effect of CP/CPPS-A-Exos and LPS-Exos in vitro and in vivo. (A)-(B) Exosomes derived from the prostatic fluid of CP/CPPS-A patients (CP/CPPS-A Exos) (A) and LPS-stimulated RWPE-1 cells (LPS-Exos) (B) were stained with PKH67 (green) and co-cultured with WPMY-1 cells at various time points (0, 1, 2, 4 h). WPMY-1 cells were stained with DiR and DAPI to track the time-dependent intracellular uptake of PKH67-labeled exosomes via confocal microscopy (Scale bar = 20 μm). (C)-(D) Semi-quantitative analysis of the corresponding PKH67 fluorescence intensities in WPMY-1 cells. (E)-(F) The relative mRNA levels and supernatant levels of inflammatory cytokines in WPMY-1 cells co-cultured with exosomes derived from prostatic fluids were measured by qPCR (E) and ELISA (F). (G)-(H) The relative mRNA levels and supernatant levels of inflammatory cytokines in WPMY-1 cells co-cultured with exosomes derived from RWPE-1 cells were measured by qPCR (G) and ELISA (H). (I) and (N) Representative H&E staining images of prostate tissue in SD rats injected with sterile PBS, prostatic fluid derived exosomes (I), and RWPE-1 cells derived exosomes (N) (Scale bar = 200 μm). (J) and (O) The histology score for the degree of inflammation in prostates treated with exosomes derived from prostatic fluids (J) and RWPE-1 cells (O). (K) and (P) The relative expression level of MCP-1 in the serum of SD rats injected with exosomes derived from prostatic fluids (K) and RWPE-1 cells (P) was measured by ELISA. (L) and (Q) The relative mRNA level of MCP-1 in SD rat’s prostate tissue treated with exosomes derived from prostatic fluids (L) and RWPE-1 cells (Q) was measured by qPCR. (M) and (R) The relative expression level of MCP-1 in SD rat’s prostate tissue treated with exosomes derived from prostatic fluids (M) and RWPE-1 cells (R) was measured by western blotting. Statistical analyses were conducted using one-way ANOVA; *P* values are indicated on each comparison (*P < 0.05, **P < 0.01, ***P < 0.001, and ****P < 0.0001), denoting statistical significance

To explore the effect of these exosomes on stromal cell, PKH67-labeled exosomes were co-cultured with stromal cell, WPMY-1, which were subsequently labeled using DiR. Immunofluorescence indicated that both the exosomes derived from the prostatic fluid of CP/CPPS-A patients and those from LPS-stimulated RWPE-1 cells were taken up by WPMY-1 cells in a time-dependent manner (Fig. 2A-D). Subsequently, the expression of inflammatory cytokines was detected by qPCR, and the cytokines levels in culture media supernatant were measured by ELISA. The results showed that the expression and supernatant levels of MCP-1 and CXCL1 were significantly elevated, whereas IL-31 and CCL11 were decreased, in WPMY-1 cells incubated with exosomes derived from the prostatic fluid of CP/CPPS-A patients, compared to those from healthy individuals (Fig. 2E and F). However, the expression of CCL3, CXCL12, IL-6, IL-7, IL-15, and IL-17A remained unchanged (Fig. S1A and S1B). Similarly, when WPMY-1 cells were incubated with exosomes derived from LPS-stimulated RWPE-1 cells, the expression and supernatant levels of MCP-1 and IL-6 were obviously increased, while the decreased levels of CCL3, CCL11, and IL-31 were observed, compared to the expression in non-stimulated (Control) WPMY-1 cells (Fig. 2G and H). The expression of IL-15, CXCL1, IL-7, CXCL12, and IL-17A also remained unchanged (Fig. S2A and S2B). These results suggest that prostatic fluid derived exosomes and LPS-stimulated RWPE-1 cells derived exosomes may promote WPMY-1 cell inflammation by increasing MCP-1 expression.

To further identify the impact of exosomes derived from CP/CPPS-A patients on inflammation in vivo, male SD rats were injected with equal amounts of either sterile PBS, exosomes derived from CP/CPPS-A patients, or exosomes derived from healthy individuals into the ventral lobe of their prostates. H&E staining revealed no obvious infiltration of inflammatory cells in male SD rats injected with PBS or exosomes derived from healthy individuals. On the contrary, there was a notable increase in inflammatory cell infiltration in male SD rats injected with exosomes derived from CP/CPPS-A patients, compared to those injected with exosomes derived from healthy individuals (Fig. 2I and J). Meanwhile, the expression level of MCP-1 was significantly elevated in both serums and prostate tissues of male SD rats injected with exosomes derived from CP/CPPS-A patients, compared to those injected with exosomes derived from healthy individuals (Fig. 2K-M). Similarly, exosomes derived from LPS-stimulated RWPE-1 cells also led to a significant increase in inflammatory cell infiltration, the serum level of MCP-1, and prostate tissue expression levels of MCP-1 in male SD rats, compared to those injected with exosomes derived from non-stimulated (Control) RWPE-1 cells (Fig. 2N-R). The inflammation score in the control group was zero (Fig. 2J and O). Taken together, these findings suggest that the proinflammatory effect of exosomes derived from the prostatic fluid of CP/CPPS-A patients and from LPS-stimulated RWPE-1 cells is closely related to the upregulation of MCP-1 in stromal cell.

### Elevated miR-203a-3p in exosomes promotes stromal cell inflammation by upregulating MCP-1 expression

**Fig. 3:**
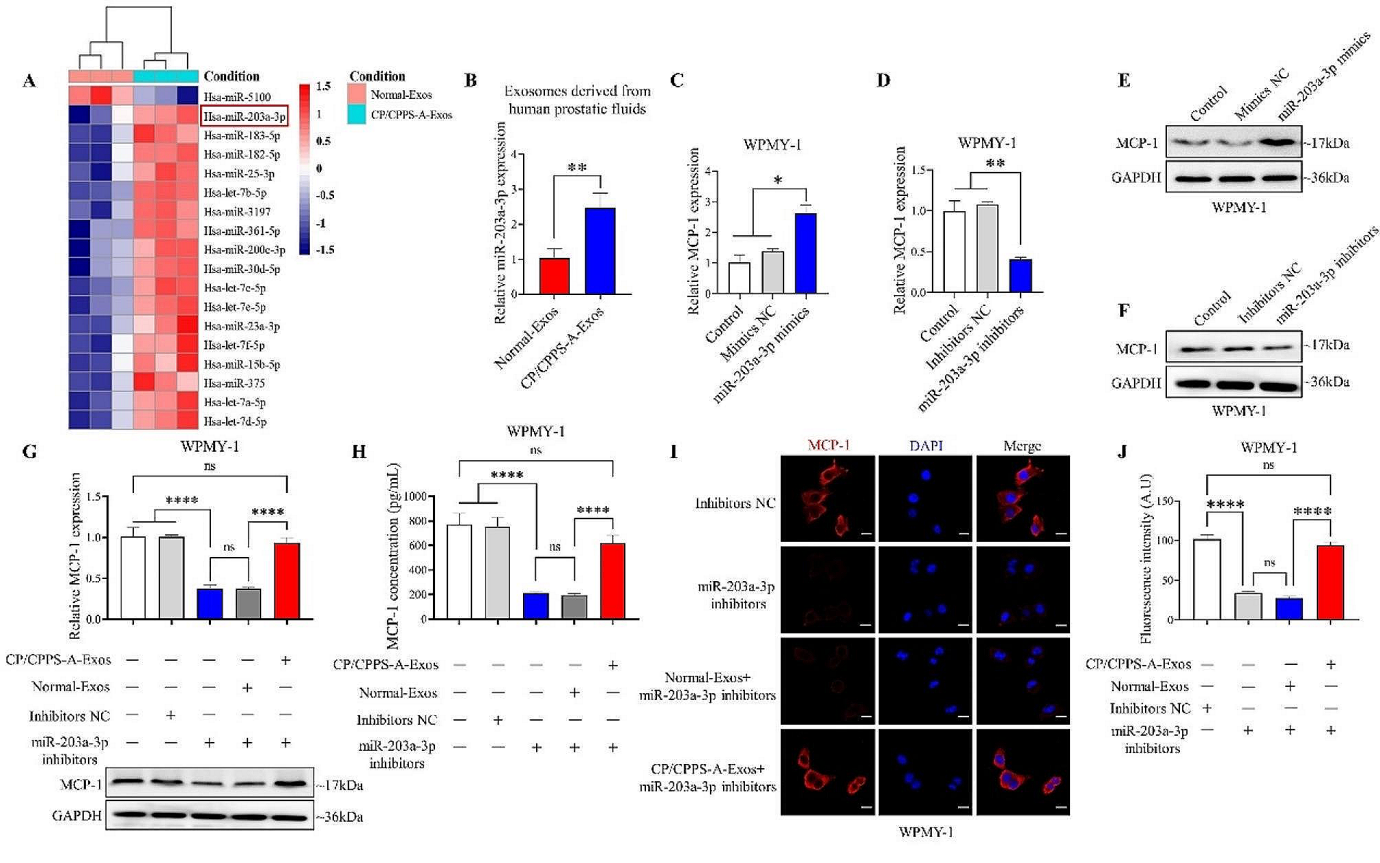
High levels of miR-203a-3p in exosomes promote stromal cell inflammation. (A) Hierarchical clustering of all differentially expressed miRNAs between exosomes derived from the prostatic fluid of healthy individuals (Normal-Exos) and CP/CPPS-A patients (CP/CPPS-A-Exos). (B) Quantification of the expression of miR-203a-3p in Normal-Exos and CP/CPPS-A-Exos, as determined by qPCR. (C)-(F) Relative mRNA (C and D) and protein (E and F) expression levels of MCP-1 in WPMY-1 cells transfected with either miR-203a-3p mimics, miR-203a-3p inhibitors, or negative controls, measured by qPCR and western blotting. (G) Relative mRNA and protein expression levels of MCP-1 in WPMY-1 cells transfected with miR-203a-3p inhibitors or controls for 8 h, then co-cultured with either Normal-Exos or CP/CPPS-A-Exos, measured by qPCR, western blotting. (H) The level of MCP-1 in the cell supernatant of WPMY-1 cells transfected with miR-203a-3p inhibitors or controls for 8 h, then co-cultured with either Normal-Exos or CP/CPPS-A-Exos, measured by ELISA. (I) Immunofluorescence staining of MCP-1 in WPMY-1 cells transfected with miR-203a-3p inhibitors or controls for 8 h, then co-cultured with either Normal-Exos or CP/CPPS-A-Exos. Visualization was done via confocal microscopy (Scale bar = 20 μm). (J) Semi-quantitative analysis of the corresponding MCP-1 fluorescence intensities in WPMY-1 cells. Statistical analyses were performed using unpaired t-tests and one-way ANOVA, with *P* values indicated on each comparison (*P < 0.05, **P < 0.01, and ****P < 0.0001), suggesting statistical significance. And with *P* values greater than or equal to 0.05 (ns ≥ 0.05) indicating a lack of statistical significance

Recent researches have shown that exosomal miRNAs play a significant role in the communication between cells [16]. Consequently, we initially analyzed the expression profile of miRNAs in exosomes derived from CP/CPPS-A patients and healthy individuals using a miRNA microarray. The results revealed that 17 miRNAs were upregulated and one miRNA was downregulated, with a fold change > 2.0, in exosomes derived from CP/CPPS-A patients compared to those from healthy individuals (Fig. 3A). Among the upregulated miRNAs, we focused on two miRNAs (let-7f-5p and miR-203a-3p) that exhibited the highest expression levels. However, previous studies have indicated that let-7f-5p has an anti-inflammatory effect in systemic lupus erythematosus [24], while miR-203a-3p has been shown to exert a proinflammatory effect [25]. Therefore, we selected miR-203a-3p for further investigation. As shown in Fig. 3B, the expression of miR-203a-3p was significantly elevated in exosomes derived from the prostatic fluid of CP/CPPS-A patients compared to those derived from healthy individuals. Moreover, stimulation with LPS significantly increased the expression of miR-203a-3p in both RWPE-1 cells and in exosomes derived from these cells (Fig. S3A and S3B). Interestingly, we observed that the expression level of miR-203a-3p was extremely higher in RWPE-1 cells compared to that in WPMY-1 cells (Fig. S3C). These results suggest that elevated expression of miR-203a-3p in inflammatory prostate epithelial cells leads to miR-203a-3p enriched in exosomes derived from prostatic fluids of CP/CPPS-A patients.

Subsequently, to elucidate whether miR-203a-3p upregulation is involved in the inflammatory behaviors of stromal cell, we examined the effect of miR-203a-3p on MCP-1 expression in WPMY-1 cells. The results revealed that miR-203a-3p mimics markedly promoted the mRNA and protein levels of MCP-1. In contrast, miR-203a-3p inhibitors exhibited the opposite effects (Fig. S3D-F and Fig. 3C-F). These results indicate that miR-203a-3p plays a proinflammatory role in stromal cell. To further explore whether exosomal miR-203a-3p derived from CP/CPPS-A patients could promote inflammation in stromal cell, we used miR-203a-3p inhibitors to decrease its expression in WPMY-1 cells and then incubated these cells with exosomes derived from either the prostatic fluid of CP/CPPS-A patients or healthy individuals. As shown in Fig. 3G-J, exosomes derived from the prostatic fluid of CP/CPPS-A patients counteracted the miR-203a-3p inhibitors-induced decrease in the expression of MCP-1 in WPMY-1 cells and the cell supernatant, while exosomes derived from healthy individuals did not cause any change. These findings suggest that exosomal miR-203a-3p derived from the prostatic fluid of CP/CPPS-A patients promotes inflammation in stromal cell by modulating MCP-1 expression.

### DUSP5 is a novel target gene of miR-203a-3p and mediates miR-203a-3p to promote MCP-1 expression

**Fig. 4:**
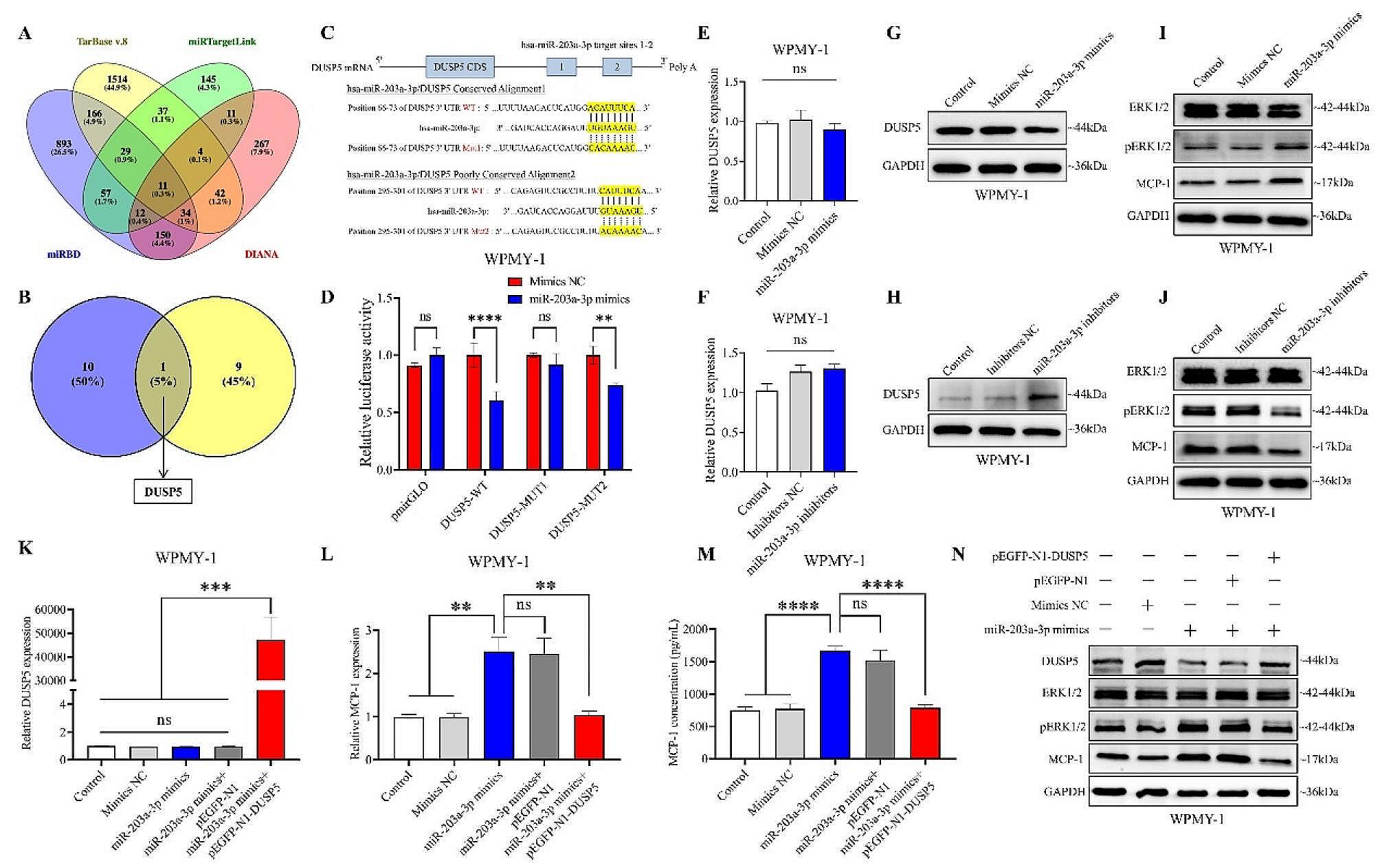
DUSP5 is a novel target gene of miR-203a-3p and mediates miR-203a-3p to promote MCP-1 expression. (A) Venn diagram illustrating the overlap of target genes for miR-203a-3p, as predicted by four databases: Tarbase v.8, miRDB, DIANA, and mirtargetlink2.0. (B) Venn diagrams showing the intersection of candidate genes derived from the 11 candidate targets in (A) and the 10 genes from miR-203a-3p involved in regulating the MAPK signaling pathway. (C) Construction of the wild-type (WT) and mutated types (MUT1, MUT2) luciferase reporter for miR-203a-3p binding sites in the full-length 3’UTR of DUSP5 mRNA. (D) The WT or mutated types (MUT1, MUT2) reporter constructs were co-transfected into WPMY-1 cells with miR-203a-3p mimics or controls. Relative luciferase activities were then measured. (E)-(H) Relative DUSP5 mRNA (E and F) and DUSP5 protein (G and H) expression levels in WPMY-1 cells transfected with miR-203a-3p mimics, miR-203a-3p inhibitors or controls were measured by qPCR and western blotting, respectively. (I)-(J) Relative ERK1/2, pERK1/2, and MCP-1 protein expression levels in WPMY-1 cells transfected with miR-203a-3p mimics, miR-203a-3p inhibitors or controls were measured by western blotting. (K)-(N) Relative DUSP5 mRNA (K), MCP-1 mRNA (L), MCP-1 in cell supernatant level (M), and DUSP5, ERK1/2, pERK1/2, MCP-1 protein (N) expression levels in WPMY-1 cells transfected with miR-203a-3p mimics or controls for 8 h, then co-transfected with pEGFP-N1-DUSP5 vector or pEGFP-N1 vector were measured by qPCR, ELISA, and western blotting. Statistical analyses were performed using one-way ANOVA and two-way ANOVA; *P* values are indicated on each comparison (**P < 0.01, ***P < 0.001, and ****P < 0.0001), denoting statistical significance. And with *P* values greater than or equal to 0.05 (ns ≥ 0.05) indicating a lack of statistical significance

It is well known that microRNA binds to the 3’UTR of the target gene and inhibits its translation, while exosomal miR-203a-3p derived from CP/CPPS-A patients promotes inflammation by upregulating MCP-1 expression, suggesting that MCP-1 may not be a direct target gene of miR-203a-3p. To investigate the potential molecular mechanism by which miR-203a-3p promotes inflammation in stromal cell, we predicted the target genes of miR-203a-3p in TarBase v.8, miRDB, DIANA, and mirtargetlink2.0. As shown in Figs. 4A, 11 candidate target genes were predicted in all four databases by Venn diagram analysis (Fig. 4A and Table S4). Moreover, our miRNA microarray data (Fig. 3A) combined with the DIANA-mirPath v3 tool showed that miR-203a-3p was involved in the regulation of the MAPK signaling pathway, which included 10 genes (Fig. S4A and Table S5). We then cross-referenced these 10 genes with the 11 candidate targets and found that DUSP5 was the only gene common to both sets (Fig. 4B).

Next, we aimed to confirm whether DUSP5 was a direct target gene of miR-203a-3p. We observed that the 3’UTR of DUSP5 mRNA had two binding sites for miR-203a-3p. Moreover, the mature sequence of miR-203a-3p is highly conserved across different species according to the miRBase database (Fig. S4B). Therefore, we constructed luciferase reporter gene plasmids containing the full length of the 3’UTR of DUSP5 either the Wild-type binding site or one of the two mutant binding sites (Fig. 4C). We found that miR-203a-3p mimics decreased the relative luciferase activity in WPMY-1 cells transfected with plasmids containing either the wild-type binding site or mutant binding site 2. However, it did not affect the relative luciferase activity in cells transfected with plasmids containing mutant binding site 1 (Fig. 4D). This suggests that miR-203a-3p directly targets the binding site 1 of DUSP5 3’UTR. Furthermore, transfected miR-203a-3p mimics and miR-203a-3p inhibitors into WPMY-1 cells did not affect the mRNA expression of DUSP5 (Fig. 4E and F), while significantly altering the protein expression of DUSP5 (Fig. 4G, Fig. S5A and 4 H). Collectively, these results indicated that DUSP5 was a novel target gene of miR-203a-3p in WPMY-1 cells.

Given that the above bioinformatics analysis showed that miR-203a-3p was involved in the regulation of the MAPK signaling pathway, and previous studies have shown that DUSP5 negatively regulates ERK signaling in a phosphatase activity-dependent manner [26, 27]. Therefore, we investigated the regulatory effect of miR-203a-3p on the ERK pathway in WPMY-1 cells. First, we transfected WPMY-1 cells with overexpressed (OE)-DUSP5 and si-DUSP5, and the protein levels of ERK1/2, pERK1/2, and MCP-1 were detected. The results showed that pERK1/2 and MCP-1 were significantly inhibited in the overexpressed DUSP5 cells, but no significant change was observed in total ERK expression (Fig. S6A). However, the expression of pERK1/2 and MCP-1 was increased in si-DUSP5 treated cells compared with that in si-NC treated cells (Fig. S6B). On the contrary, miR-203a-3p mimics obviously increased pERK1/2 and MCP-1, but miR-203a-3p inhibitors suppressed ERK phosphorylation and MCP-1 expression (Fig. 4I and J). To further identify the role of DUSP5 in miR-203a-3p promoting ERK phosphorylation and MCP-1 expression, we overexpressed DUSP5 in miR-203a-3p mimics treated WPMY-1 cells (Fig. 4K). As shown in Fig. 4L and M, overexpression of DUSP5 significantly reduced miR-203a-3p-induced mRNA and protein expression of MCP-1, which was consistent with the phosphorylation of ERK1/2 and the protein levels of MCP-1 in WPMY-1 cells (Fig. 4N and Fig. S5B). These findings suggest that miR-203a-3p promotes inflammation in stromal cell by regulating the DUSP5-ERK1/2-MCP-1 signaling pathway.

### PECs-derived exosomal miR-203a-3p promotes stromal cell inflammation via regulating DUSP5-ERK1/2-MCP-1 axis in vitro

**Fig. 5:**
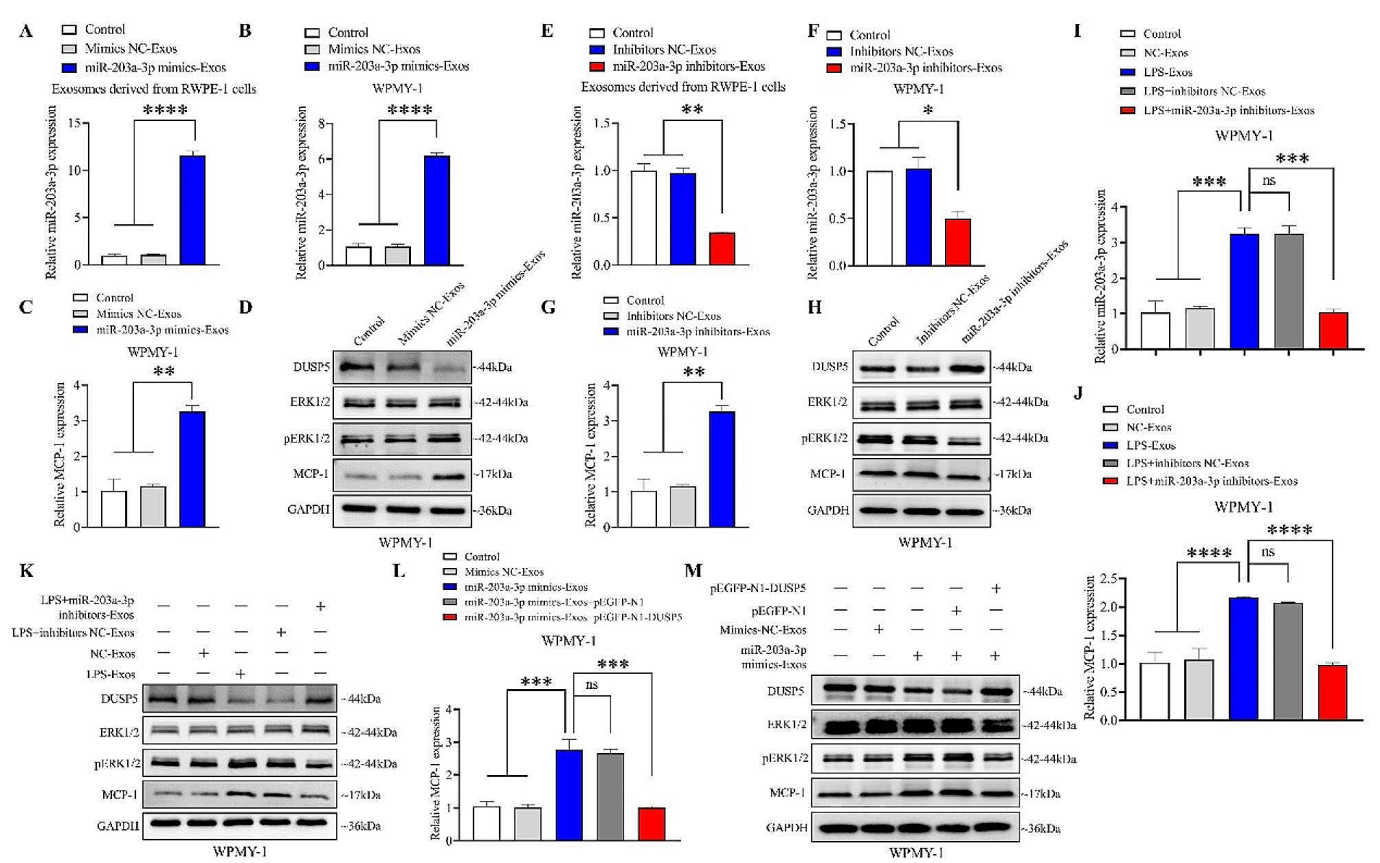
PECs-derived exosomal miR-203a-3p promotes stromal cell inflammation via regulating DUSP5-ERK1/2-MCP-1 axis in vitro. (A) Relative expression levels of miR-203a-3p in exosomes derived from RWPE-1 cells treated with miR-203a-3p mimics or controls, as determined by qPCR. (B)-(C) Relative expression levels of miR-203a-3p (B) and MCP-1 (C) in WPMY-1 cells co-cultured with exosomes derived from RWPE-1 cells transfected with miR-203a-3p mimics (miR-203a-3p mimics-Exos) or controls, as determined by qPCR. (D) The protein expression levels of DUSP5, ERK1/2, pERK1/2, and MCP-1 in WPMY-1 cells cultured with miR-203a-3p mimics-Exos or controls, measured by western blotting. (E) Relative expression levels of miR-203a-3p in exosomes derived from RWPE-1 cells treated with miR-203a-3p inhibitors or controls, as determined by qPCR. (F)-(G) Relative expression levels of miR-203a-3p (F) and MCP-1 (G) in WPMY-1 cells cultured with exosomes derived from RWPE-1 cells transfected with miR-203a-3p inhibitors (miR-203a-3p inhibitors-Exos) or controls, as determined by qPCR. (H) The protein expression levels of DUSP5, ERK1/2, pERK1/2, and MCP-1 in WPMY-1 cells cultured with miR-203a-3p inhibitors-Exos or controls, measured by western blotting. (I)-(K) Relative RNA expression levels of miR-203a-3p (I) and MCP-1 (J), and protein expression levels of DUSP5, ERK1/2, pERK1/2, MCP-1(K) in WPMY-1 cells cultured with LPS-Exos, LPS + miR-203a-3p inhibitors-Exos or controls, measured by qPCR and western blotting, respectively. (L)-(M) Relative expression levels of MCP-1 mRNA (L) and DUSP5, ERK1/2, pERK1/2, MCP-1 protein (M) in WPMY-1 cells cultured with miR-203a-3p mimics-Exos or controls and then transfected with pEGFP-N1-DUSP5 or a control, measured by qPCR and western blotting, respectively. Statistical analyses were performed using one-way ANOVA, with *P* values indicated on each comparison (*P < 0.05, **P < 0.01, ***P < 0.001, and ****P < 0.0001), suggesting statistical significance. And with *P* values greater than or equal to 0.05 (ns ≥ 0.05) indicating a lack of statistical significance

To investigate whether exosomal miR-203a-3p derived from PECs could regulate the DUSP5-ERK1/2-MCP-1 signaling pathway, miR-203a-3p mimics or inhibitors were transfected into RWPE-1 cells, exosomes derived from transfected RWPE-1 cells were collected and the expression of miR-203a-3p in RWPE-1 cells and in exosomes derived from RWPE-1 was detected. qPCR results showed that miR-203a-3p mimics or inhibitors were successfully transferred into RWPE-1 cells (Fig. S7A and S7B), and miR-203a-3p was also increased in RWPE-1 cell derived exosomes (Fig. 5A). Furthermore, stromal cell WPMY-1 incubated with exosomes derived from RWPE-1 cells showed an elevated level of miR-203a-3p (Fig. 5B). Consistently, the increased mRNA and protein expression of MCP-1 were observed in WPMY-1 cells, which were co-cultured with exosomes derived from miR-203a-3p transfected RWPE-1 cells (Fig. 5C and D). Exosomes containing overexpression of miR-203a-3p induced ERK1/2 phosphorylation by decreasing DUSP5 protein expression in WPMY-1 cells (Fig. 5D). Conversely, inhibition of miR-203a-3p in epithelial cells resulted in the opposite effects in WPMY-1 cells (Fig. 5E-H).

To further demonstrate whether epithelial cells derived exosomal miR-203a-3p under inflammatory condition promotes stromal cell inflammation, miR-203a-3p inhibitors were used in LPS-stimulated RWPE-1 cells. Then WPMY-1 cells were incubated with these exosomes. Exosomes derived from LPS-stimulated RWPE-1 cells upregulated miR-203a-3p and MCP-1 expression in WPMY-1 cells, which was compared with exosomes derived from RWPE-1 cells without LPS-stimulation. However, exosomes derived from LPS-stimulated RWPE-1 cells transfected with miR-203a-3p inhibitors remarkably decreased the expression of miR-203a-3p and MCP-1 (Fig. 5I and J). Consistent with these results, western blotting analysis showed corresponding changes in the protein levels of DUSP5, pERK1/2, and MCP-1 (Fig. 5K). Meanwhile, we observed that when WPMY-1 cells incubated with exosomes transfected with miR-203a-3p mimics-Exos, overexpression of DUSP5 significantly attenuated the induction effect of miR-203a-3p on MCP-1 expression (Fig. 5L and M). Moreover, the protein levels of DUSP5 and pEKR1/2 in WPMY-1 cells also changed accordingly (Fig. 5M). These data suggest that exosomal miR-203a-3p, derived from LPS-stimulated PECs, promotes inflammation in stromal cell by regulating the DUSP5-ERK1/2-MCP-1 axis.

### Exosomes derived from PECs effectively deliver miR-203a-3p antagomirs to the prostate

**Fig. 6:**
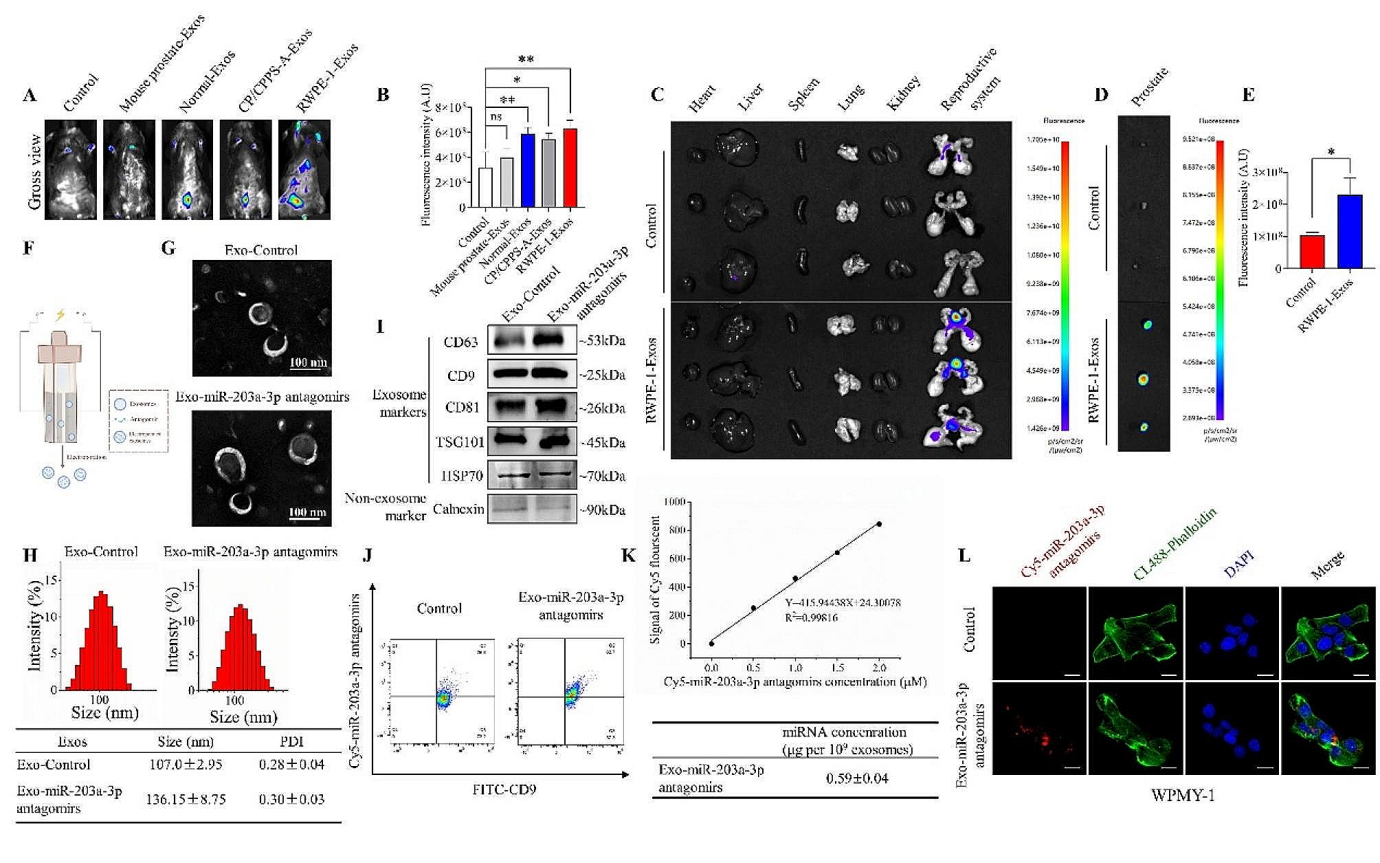
Exosomes derived from RWPE-1 cells effectively deliver miR-203a-3p antagomirs to the prostate. (A) Approximately 100 µg (at protein level) in 100 µL of exosomes derived from the prostatic fluid of healthy individuals, the prostatic fluid of CP/CPPS-A patients, mouse prostate tissues, and RWPE-1 cells were labeled with PKH67 and injected into normal C57BL/6 mice via the tail vein. Roughly 24 h after the injection, in vivo fluorescence imaging was performed. Representative fluorescence images are shown for at least 3 mice in each group. (B) Quantification data representing the relative fluorescence intensity of infiltrated exosomes in the lower abdomen of C57BL/6 mice in different groups. (C)-(D) Major organs, including heart, liver, spleen, lung, kidney, reproductive system, and prostate, were harvested for ex vivo imaging. (E) Quantification data representing the relative fluorescence intensity of infiltrated exosomes in the prostate of C57BL/6 mice in different groups. (F) A scheme illustrating the preparation of miR-203a-3p antagomirs loaded exosomes. (G) Representative images of exosomal morphology observed via TEM (Scale bar = 100 nm). (H) NTA results showed the diameter of exosomes. (I) Western blotting results demonstrating the presence of exosome markers. (J) Cy5-miR-203a-3p antagomirs loaded exosomes were labeled with FITC-CD9 and analyzed on a BD FACS cantoII. (K) A standard curve for Cy5-miR-203a-3p antagomirs was created using the Microplate Reader method (detection at 650/670 nm), and quantification of Cy5-miR-203a-3p antagomirs loaded exosomes was performed. (L) Cy5-miR-203a-3p antagomirs loaded exosomes (red) were co-cultured with WPMY-1 cells. WPMY-1 cells were stained with CL488-Phalloidin and DAPI to observe the uptake of electroporated exosomes by confocal microscopy (Scale bar = 20 μm). Statistical analyses were performed using unpaired t-tests and one-way ANOVA, with *P* values indicated on each comparison (*P < 0.05 and **P < 0.01), suggesting statistical significance. And with *P* values greater than or equal to 0.05 (ns ≥ 0.05) indicating a lack of statistical significance

To detect whether exosomes could serve as a delivery system for miR-203a-3p antagomirs to the prostate, we labeled exosomes derived from CP/CPPS-A patients, healthy subjects, RWPE-1 cells, and mouse prostatic tissues with PKH67. Upon intravenous administration of exosomes into C57BL/6 mice, we found that exosomes could be delivered to the lower abdomen, and the fluorescence intensity of exosomes derived from RWPE-1 cells was significantly higher than that from other sources (Fig. 6A and B). Interestingly, the fluorescence intensities of RWPE-1 cells derived exosomes in the heart, liver, spleen, lung, and kidney were lower than reproductive system, especially in the prostate (Fig. 6C-E). These results further demonstrated that RWPE-1 cells derived exosomes had the ability to target the prostate.

Subsequently, we loaded miR-203a-3p antagomirs into exosomes derived from RWPE-1 cells by using electroporation (Fig. 6F). TEM revealed no significant change in the characteristic bowl shape between miR-203a-3p antagomirs-loaded exosomes and control exosomes (Fig. 6G). NTA showed that miR-203a-3p antagomirs-loaded exosomes and control exosomes had average diameters of 107.0 ± 2.95 nm and 136.2 ± 8.75 nm, respectively (Fig. 6H). CD9, CD63, CD81, TSG101, and HSP70 were present in antagomirs-loaded exosomes, but no Calnexin was present in the miR-203a-3p antagomirs-loaded exosomes (Fig. 6I). These results suggested that the biological function of the exosomes was not affected by electroporation. The encapsulation efficiency of Cy5-labeled miR-203a-3p antagomirs in RWPE-1-derived exosomes, as measured by flow cytometry, was found to be 62.7%. The flow cytometry result of the respective control was found to be 26.8% (Fig. 6J and Fig. S8). The loading capacity of miR-203a-3p antagomirs in 10^9^ exosomes was determined to be 0.59 ± 0.04 µg (Fig. 6K). Furthermore, the exosomes containing Cy5-labeled miR-203a-3p antagomirs were taken up by WPMY-1 cells (Fig. 6L). Taken together, these data suggest that exosomes derived from RWPE-1 cells could potentially serve as an effective vehicle for the delivery of miR-203a-3p antagomirs to the prostate in vivo.

### Exosomes derived from PECs encapsulating miR-203a-3p antagomirs alleviate prostatitis in male C57BL/6 mice

**Fig. 7:**
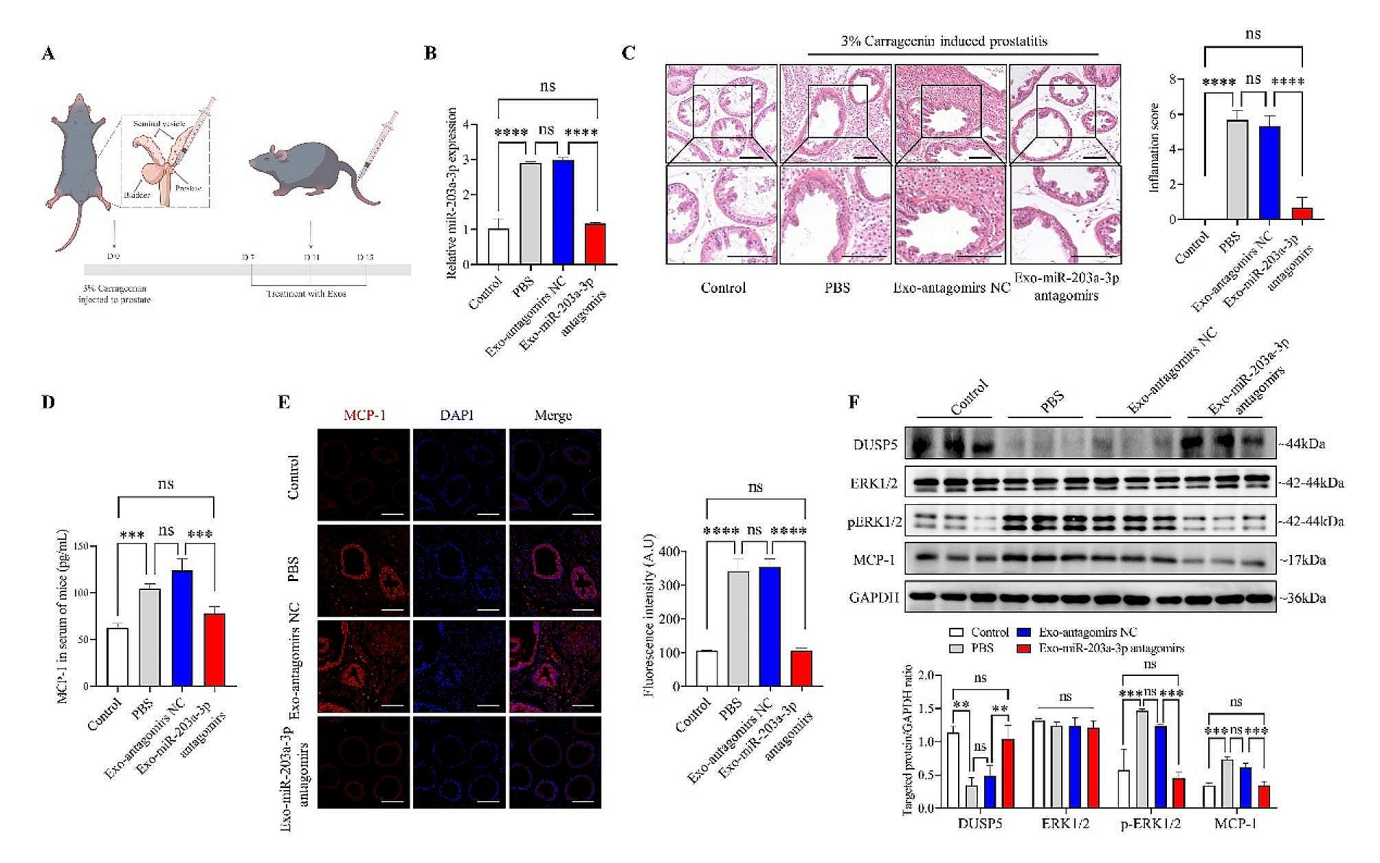
The anti-inflammatory effect of exosomes derived from RWPE-1 cells loaded with miR-203a-3p antagomirs in vivo. (A) A scheme illustrating the protocol for intravenous injection of Exo-miR-203a-3p antagomirs every 3 days, with a total of 3 times injections per mouse, following the injection of 3% carrageenan into the ventral lobes of the prostate gland. (B) Expression levels of miR-203a-3p in prostate tissues from different groups, as determined by qPCR. (C) H&E staining images of prostate tissue and inflammation scores for the prostate in different groups (Scale bar = 200 μm). (D) Quantification of MCP-1 expression in the serum of mice by ELISA. (E) Immunofluorescence staining and quantification of the fluorescence intensity of MCP-1 in prostate tissues of mice from different groups (Scale bar = 200 μm). (F) The expression of DUSP5, ERK1/2, pERK1/2, and MCP-1 in mice prostates, as measured by western blotting. Lower panel showed the expression normalized to that of GAPDH. Statistical analyses were performed using one-way ANOVA, with *P* values indicated on each comparison (***P < 0.001 and ****P < 0.0001), suggesting statistical significance. And with *P* values greater than or equal to 0.05 (ns ≥ 0.05) indicating a lack of statistical significance

To further confirm the therapeutic effects of exosomes derived from RWPE-1 cells loaded with miR-203a-3p antagomirs, the exosomes were administered to the chronic prostatitis mice by intravenous injection (Fig. 7A). We found that the exosomes loaded with miR-203a-3p antagomirs significantly reduced the expression of miR-203a-3p in the prostate tissues (Fig. 7B) and extremely reduced the infiltration of inflammatory cells in prostate tissue (Fig. 7C). Moreover, the serum levels of MCP-1 were significantly decreased in the exosomes loaded with the Exo-miR-203a-3p antagomirs group compared with the exosomes loaded with the Exo-antagomirs NC control group (Fig. 7D). Importantly, immunofluorescence (Fig. 7E) and western blotting (Fig. 7F) assay confirmed that exosomes loaded with miR-203a-3p antagomirs downregulated the expression of MCP-1 in prostate tissue, which was due to that DUSP5 expression was up-regulated by miR-203a-3p antagomirs, thereby inhibiting the phosphorylation of ERK1/2. Collectively, our data revealed that exosomes loaded with miR-203a-3p antagomirs could target the prostate and attenuate inflammation, and the DUSP5-ERK1/2-MCP-1 pathway played an important role in the treatment of prostatitis with the exosomes, suggesting that this pathway may be a novel target for CP/CPPS-A therapy.

## Discussion

**Fig. 8:**
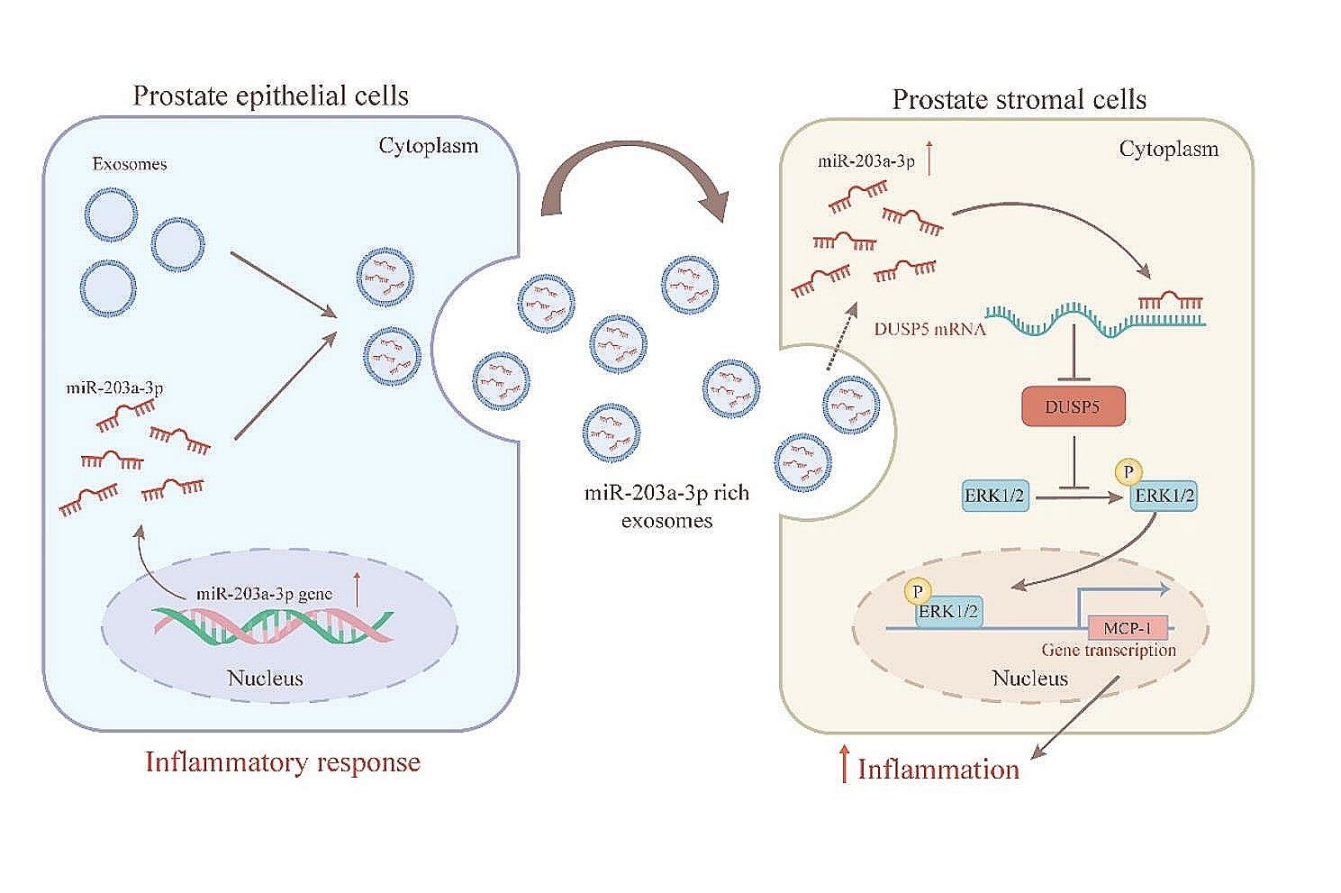
A scheme visually represents how exosomal miR-203a-3p, originating from inflammatory prostate epithelial cells, activates the ERK1/2-MCP-1 signaling pathways. This activation occurs by decreasing the expression of DUSP5, which further promotes inflammation in stromal cell

The mechanism of inflammatory progress in the CP/CPPS-A patients remains elusive. In this study, we demonstrated that exosomes derived from the prostatic fluid of CP/CPPS-A patients promoted inflammation in stromal cell through high levels of miR-203a-3p in the exosomes. Subsequently, we clarified that the proinflammatory mechanism of miR-203a-3p was to promote MCP-1 expression by regulating the DUSP5-ERK1/2 pathway (Fig. 8). Particularly, we developed miR-203a-3p antagomirs-loaded exosomes derived from RWPE-1 cells and confirmed their therapeutic effects in a mouse model of chronic prostatitis. These findings provide new insights into the pathogenesis of inflammation in CP/CPPS-A and suggest potential therapeutic strategies for this condition.

PECs and PSCs are the two major cell types in prostate tissue, each with distinct functions that are essential for normal prostate function [28]. In CP/CPPS patients, PECs may change due to inflammation, which disrupts the epithelial barrier and allows potentially harmful substances, including bacteria, to penetrate the prostate tissue [29]. This infiltration could exacerbate the inflammation and pain commonly associated with CP/CPPS. PSCs provide structural support to the prostate gland and also produce various signaling molecules that can influence the behavior of other cells within the prostate [11]. In CP/CPPS, PSCs may contribute to the inflammatory response and the development of fibrosis (the thickening and scarring of connective tissue), which contributes to the symptoms of the disease [7]. However, the communication between PECs and PSCs in the context of CP/CPPS-A remains largely unexplored. Our study sheds light on the role of exosomes derived from the prostatic fluid of CP/CPPS-A patients in mediating inflammation in stromal cell. This finding highlights a potential mechanism of communication between PECs and PSCs, accumulating data for further understanding the inflammatory progression of CP/CPPS-A.

Exosomes, which serve as a tool for intercellular communication, have been reported to play a critical role in inflammation. Previous studies have shown that urine exosomes in CP/CPPS-A patients are closely related to prostate cancer [30]. However, the specific role of exosomes derived from the prostatic fluid of CP/CPPS-A patients remains largely unclear. Our study showed that exosomes derived from the prostatic fluid of CP/CPPS-A patients could mediate inflammation in the stroma of the prostate, as evidenced by the infiltration of inflammatory cells and upregulation of MCP-1 in male SD rats. This finding underscored the importance of exosomes in the context of CP/CPPS-A and suggested that their contents were vital in exerting their biological functions. Exosomes could carry a variety of molecules, including proteins, lipids, and nucleic acids, thereby affecting various physiological and pathological processes of recipient cells [31]. Of particular interest is exosomal miRNAs, which have been shown to play crucial roles in regulating the biological functions of recipient cells. It is well known that the function of miRNAs is to post-transcriptionally regulate the expression of target genes. When packaged into exosomes, miRNAs can be transferred from one cell to another, effectively altering the function and behavior of the recipient cells [32]. In this study, we examined the miRNA expression profile in the prostatic fluid of CP/CPPS-A patients by using a miRNA microarray, revealing that miR-203a-3p was the most significantly upregulated miRNA. This finding suggested that miR-203a-3p could be a key player in the pathogenesis of CP/CPPS-A and further highlighted the importance of exosomal miRNAs in the processes of diseases.

Previous studies have confirmed that miR-203a-3p is involved in the inflammatory process of several diseases. For example, miR-203a is involved in HBx-induced inflammation by targeting Rap1a [33]. However, our results showed that the expression of miR-203a-3p was lower in stromal cell, but was highly expressed in exosomes derived from the prostatic fluid of CP/CPPS-A patients and LPS-stimulated epithelial cells. The inflammatory progress of stromal cell was derived by exosomal miR-203a-3p. Moreover, the homology of miR-203a-3p across humans, rats, and mice indicates that the biological functions of exosomal miR-203a-3p, whether derived from patient samples or RWPE-1 cells, may be conserved across different species. Therefore, the findings from our SD rat model and C57BL/6 mouse model could potentially apply to human cases of CP/CPPS-A.

DUSP5 is a member of proteins known as dual-specificity phosphatases (DUSPs), which are enzymes that can remove phosphate groups from both tyrosine and serine/threonine residues on other proteins [34]. DUSP5 is particularly important in regulating the activity of mitogen-activated protein kinases (MAPKs), which are involved in transmitting signals from the cell surface to the DNA in the cell nucleus [35]. Previous studies have demonstrated that DUSP5 is particularly important in regulating the activity of mitogen-activated protein kinases (MAPKs), which can specifically inactivate the key protein of MAPK pathway ERK1/2 [36]. Moreover, activated ERK1/2 can induce the production of proinflammatory cytokines and chemokines, which can recruit immune cells to the prostate and exacerbate inflammation [37, 38]. Our findings revealed that exosomal miR-203a-3p, derived from the prostatic fluid of CP/CPPS-A patients and inflammatory PECs, directly targeted DUSP5 and reduced the protein level of DUSP5, subsequently promoting stromal cell inflammation by activating ERK1/2 phosphorylation and MCP-1 expression. It also indicates that the DUSP5-ERK1/2 pathway plays an important role in stromal cell inflammation induced by exosomal miR-203a-3p.

Interestingly, consistent with the fact that exosomes can be used as drug delivery systems [39–42], we discovered that exosomes derived from RWPE-1 cells could serve as an effective delivery system for miR-203a-3p antagomirs in a mouse model of chronic prostatitis. Moreover, we confirmed that exosomes derived from RWPE-1 cells have an optimal targeting ability for the prostate, compared with exosomes derived from mouse prostate tissues, the prostatic fluid of CP/CPPS-A patients, and the prostatic fluid of healthy individuals. This specificity could be attributed to the fact that exosomes derived from RWPE-1 cells were more purified compared to others. The other samples, such as those from mouse prostate tissues and the prostatic fluid of both CP/CPPS-A patients and healthy individuals, contain multiple types of cells, which could result in a more heterogeneous population of exosomes. However, it is not yet clear whether exosomes derived from RWPE-1 cells can be used as a viable drug delivery system for CP/CPPS-A patients. Further research is needed to determine its effectiveness.

Overall, our study sheds light on the molecular mechanisms underlying CP/CPPS-A associated inflammation process and suggests a promising approach for the development of targeted therapies. These findings have the potential to benefit individuals suffering from this challenging condition by providing new avenues for treatment and management.

### Electronic supplementary material

Below is the link to the electronic supplementary material.

Supplementary Material 1

## Data Availability

No datasets were generated or analysed during the current study.
