# Supplementary material for: Epithelial cells derived exosomal miR-203a-3p facilitates stromal inflammation of type IIIA chronic prostatitis/chronic pelvic pain syndrome by targeting DUSP5 and increasing MCP-1 generation
Source: J Nanobiotechnology. 2024 May 10;22:236. doi: 10.1186/s12951-024-02513-5 (PMC11084011; doi:10.1186/s12951-024-02513-5)
Supplement: Supplementary file 1 — Supplementary Material 1 [file 12951_2024_2513_MOESM1_ESM.docx]

**Epithelial cells derived exosomal miR-203a-3p facilitates stromal inflammation of type IIIA chronic prostatitis/chronic pelvic pain syndrome by targeting DUSP5 and increasing MCP-1 generation**

**Supplementary Materials**


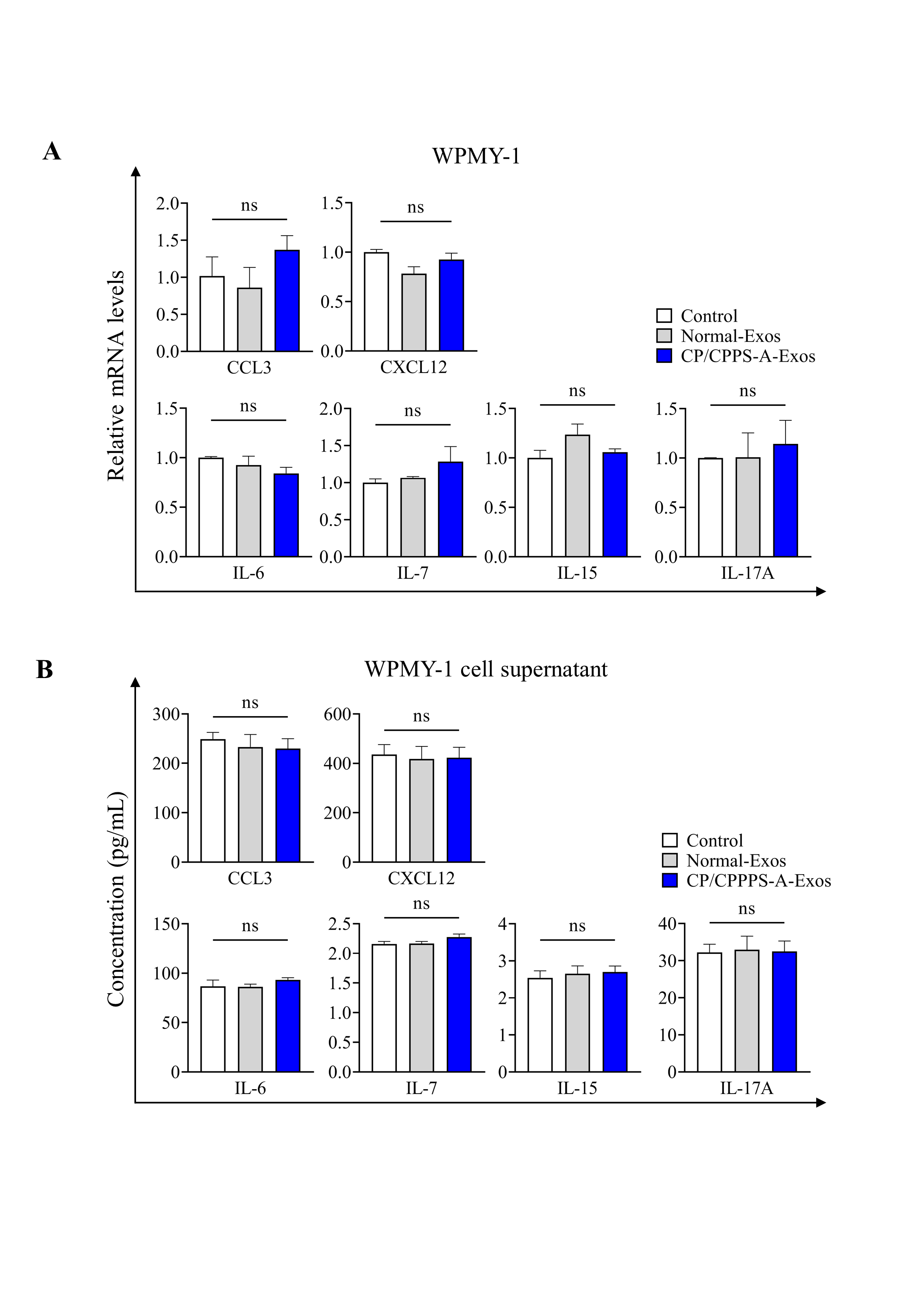


**Fig. S1 The expression and supernatant levels of inflammatory cytokines in WPMY-1 cells treated with exosomes derived from prostatic fluids**

(A) The relative mRNA levels of inflammatory cytokines in WPMY-1 cells co-cultured with exosomes derived from prostatic fluids were measured by qPCR. (B) The supernatant levels of inflammatory cytokines in WPMY-1 cells co-cultured with exosomes derived from prostatic fluids were measured by ELISA. Statistical analyses were performed using one-way ANOVA, with *P* values greater than or equal to 0.05 (ns ≥0.05) indicating a lack of statistical significance.


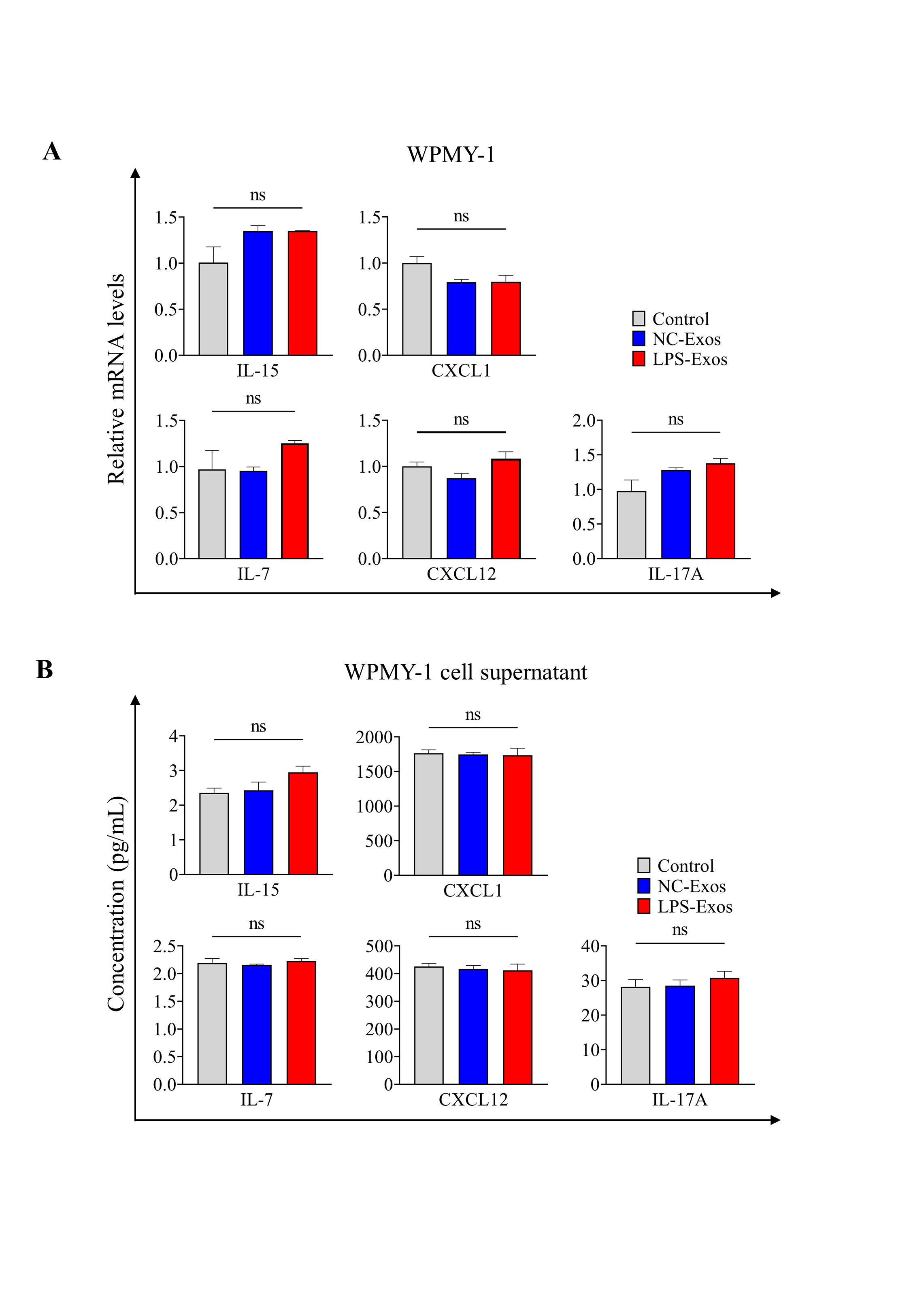


**Fig. S2 The expression and supernatant levels of inflammatory cytokines in WPMY-1 cells treated with exosomes derived from RWPE-1 cells**

(A) The relative mRNA levels of inflammatory cytokines in WPMY-1 cells co-cultured with exosomes derived from RWPE-1 cells were measured by qPCR. (B) The supernatant levels of inflammatory cytokines in WPMY-1 cells co-cultured with exosomes derived from RWPE-1 cells were measured by ELISA. Statistical analyses were performed using one-way ANOVA, with *P* values greater than or equal to 0.05 (ns ≥0.05) indicating a lack of statistical significance.


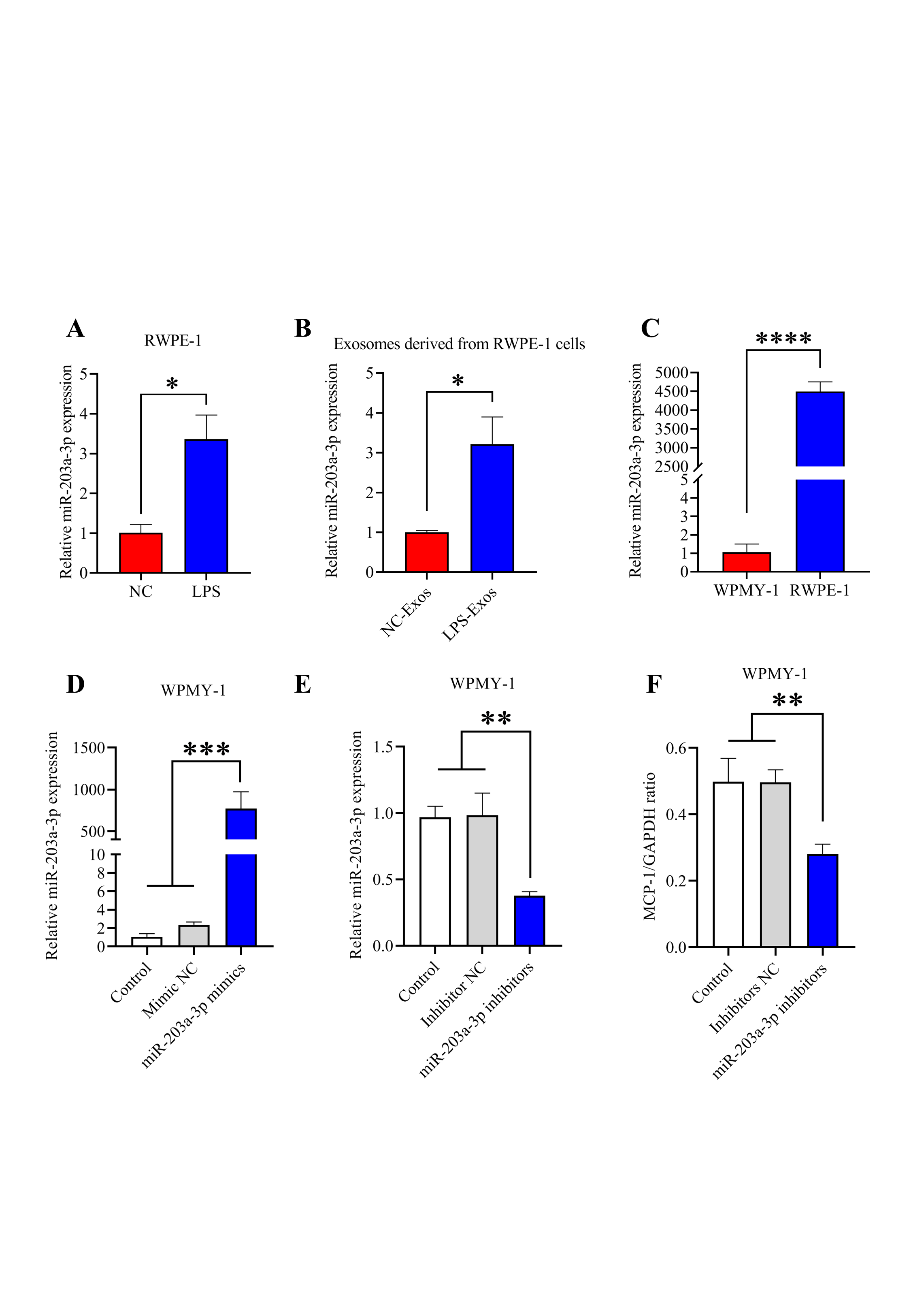


**Fig. S3 The expression of miR-203a-3p in epithelial cells and stromal cells under different conditions**

(A) Relative expression levels of miR-203a-3p in RWPE-1 cells treated with 100 ng/mL LPS or a control. (B) Relative expression levels of miR-203a-3p in exosomes derived from RWPE-1 cells treated with 100 ng/mL LPS or a control. (C) Relative expression levels of miR-203a-3p in WPMY-1 cells and RWPE-1 cells. (D) Relative expression levels of miR-203a-3p in WPMY-1 cells transfected with miR-203a-3p mimics or controls. (E) Relative expression levels of miR-203a-3p in WPMY-1 cells transfected with miR-203a-3p inhibitors or controls. (F) The expression of MCP-1 normalized to that of GAPDH in WPMY-1 cells transfected with miR-203a-3p inhibitors or negative controls, as measured by western blotting. Statistical analyses were performed using unpaired t-tests and one-way ANOVA, with *P* values indicated on each comparison (**P*<0.05, ***P*<0.01, ****P*<0.001, and *****P*<0.0001), suggesting statistical significance.


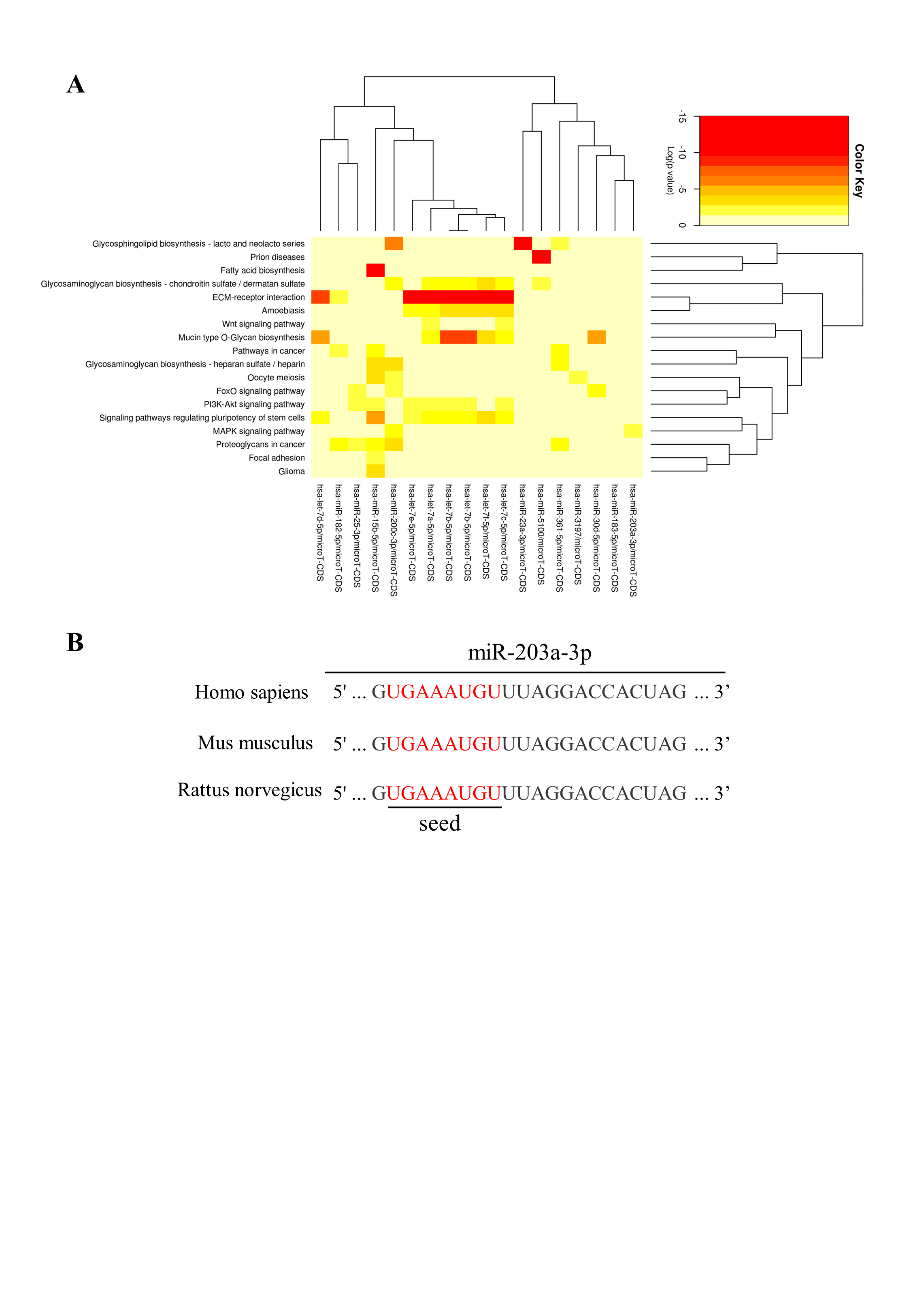


**Fig. S4 The KEGG pathways analysis and miRNA sequence alignments of miR-203a-3p**

(A) A heatmap, generated by the DIANA-miRPath v3 tool, illustrated the pathways that interacted with differentially abundant exosomal miRNAs in prostatic fluids. Orange and red colors represent lower enrichment *P* values, while shades of yellow represent higher enrichment *P* values. (B) miRNA sequence alignments of miR-203a-3p conserved in Homo sapiens, Mus musculus, and Rattus norvegicus. The seed sequence of miR-203a-3p is highlighted in red.


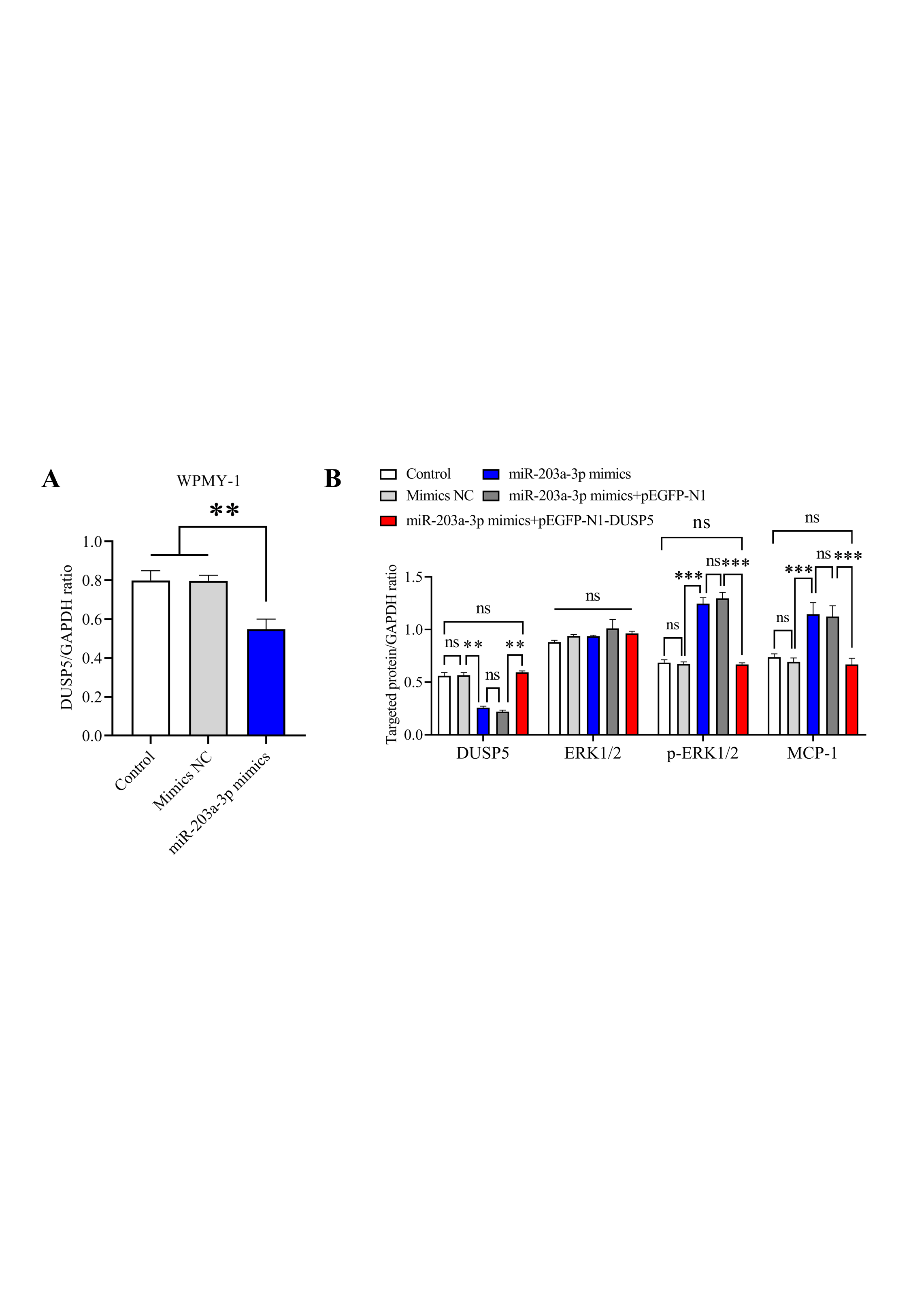


**Fig. S5 DUSP5 is a novel target gene of miR-203a-3p and mediates miR-203a-3p to promote MCP-1 expression**

(A) The expression of DUSP5 normalized to that of GAPDH in WPMY-1 cells transfected with miR-203a-3p mimics or negative controls, as measured by western blotting. (B) The expression of DUSP5, ERK1/2, pERK1/2, and MCP-1 normalized to that of GAPDH in WPMY-1 cells transfected with miR-203a-3p mimics or controls for 8 h, then co-transfected with pEGFP-N1-DUSP5 vector or pEGFP-N1 vector were measured by western blotting. Statistical analyses were performed using one-way ANOVA, *P* values are indicated on each comparison (***P*<0.01 and ****P*<0.001), denoting statistical significance. And with *P* values greater than or equal to 0.05 (ns ≥0.05) indicating a lack of statistical significance.


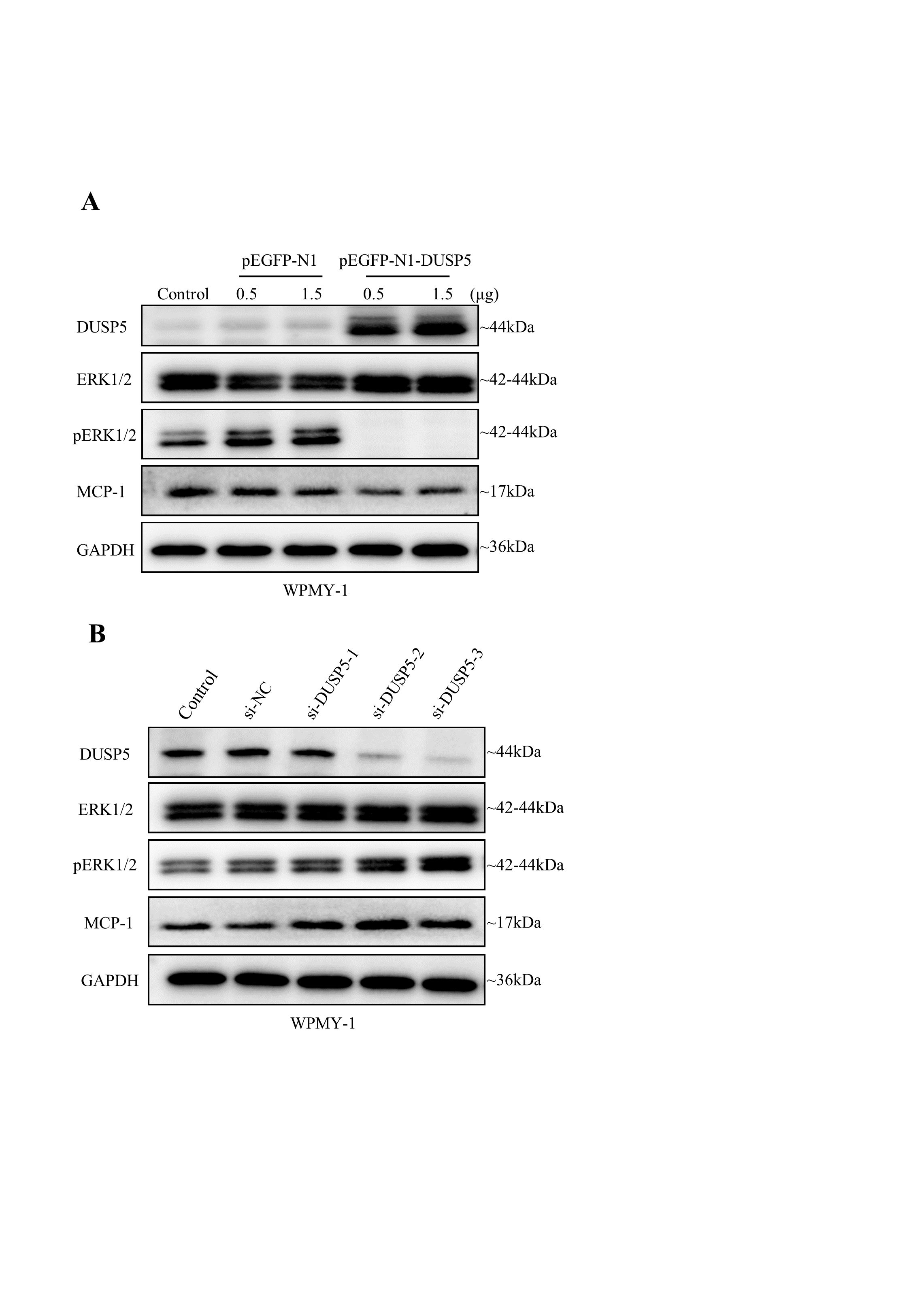


**Fig. S6 Effects of overexpressing or silencing DUSP5 in WPMY-1 cells**

(A) Relative protein expression levels of DUSP5, ERK1/2, pERK1/2, and MCP-1 in WPMY-1 cells transfected with a DUSP5 overexpression plasmid at different concentrations or a control, as detected by western blotting. (B) Relative protein expression levels of DUSP5, ERK1/2, pERK1/2, and MCP-1 in WPMY-1 cells transfected with DUSP5 silencing sequences or controls, as detected by western blotting.


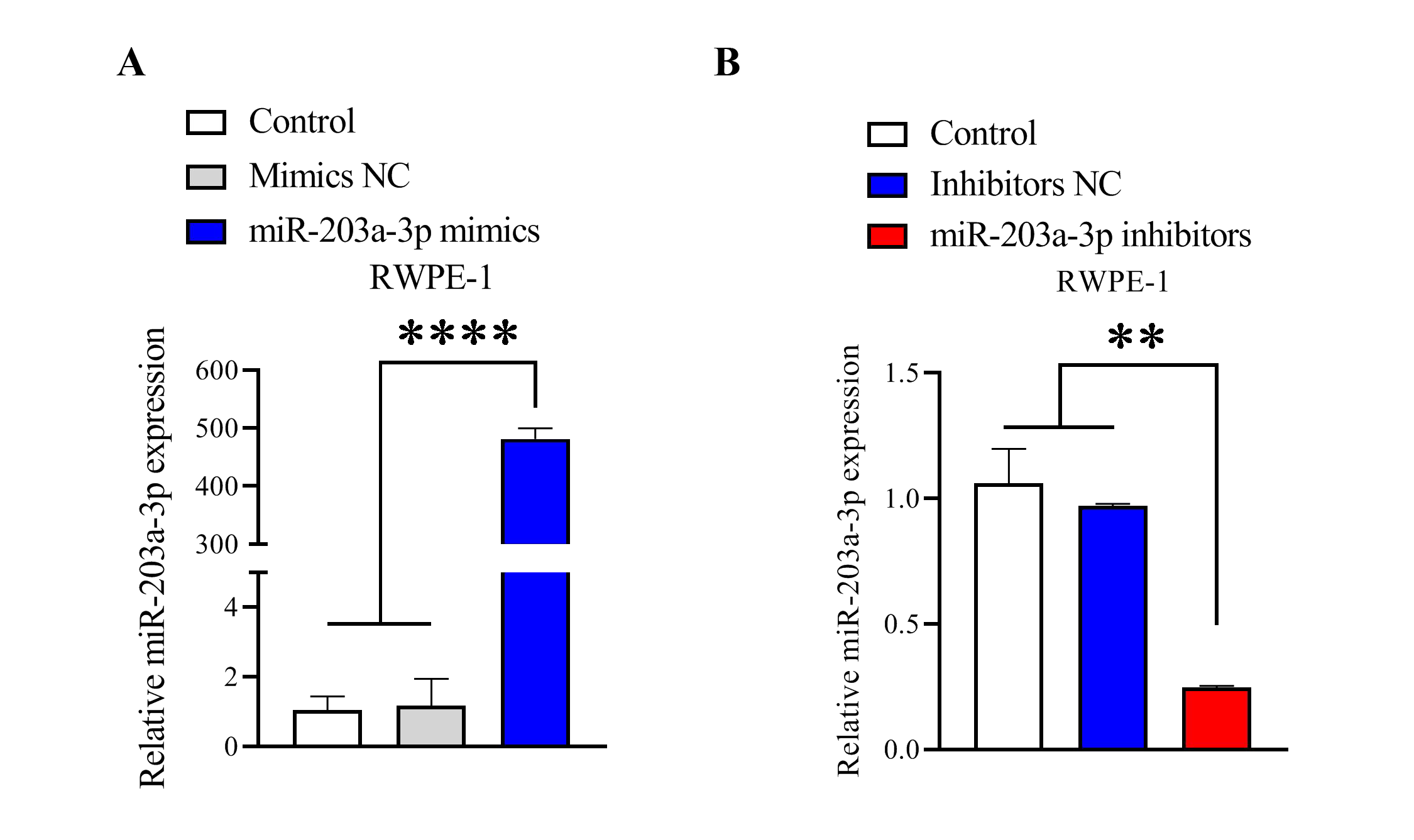


**Fig. S7 Effects of miR-203a-3p mimics or inhibitors on miR-203a-3p expression in RWPE-1 cells**

(A) Relative expression levels of miR-203a-3p in RWPE-1 cells transfected with miR-203a-3p mimics or controls, as measured by qPCR. (B) Relative expression levels of miR-203a-3p in RWPE-1 cells transfected with miR-203a-3p inhibitors or controls, measured by qPCR. Statistical analyses were performed using one-way ANOVA, with *P* values indicated on each comparison (***P*<0.01 and *****P*<0.0001) suggesting statistical significance.


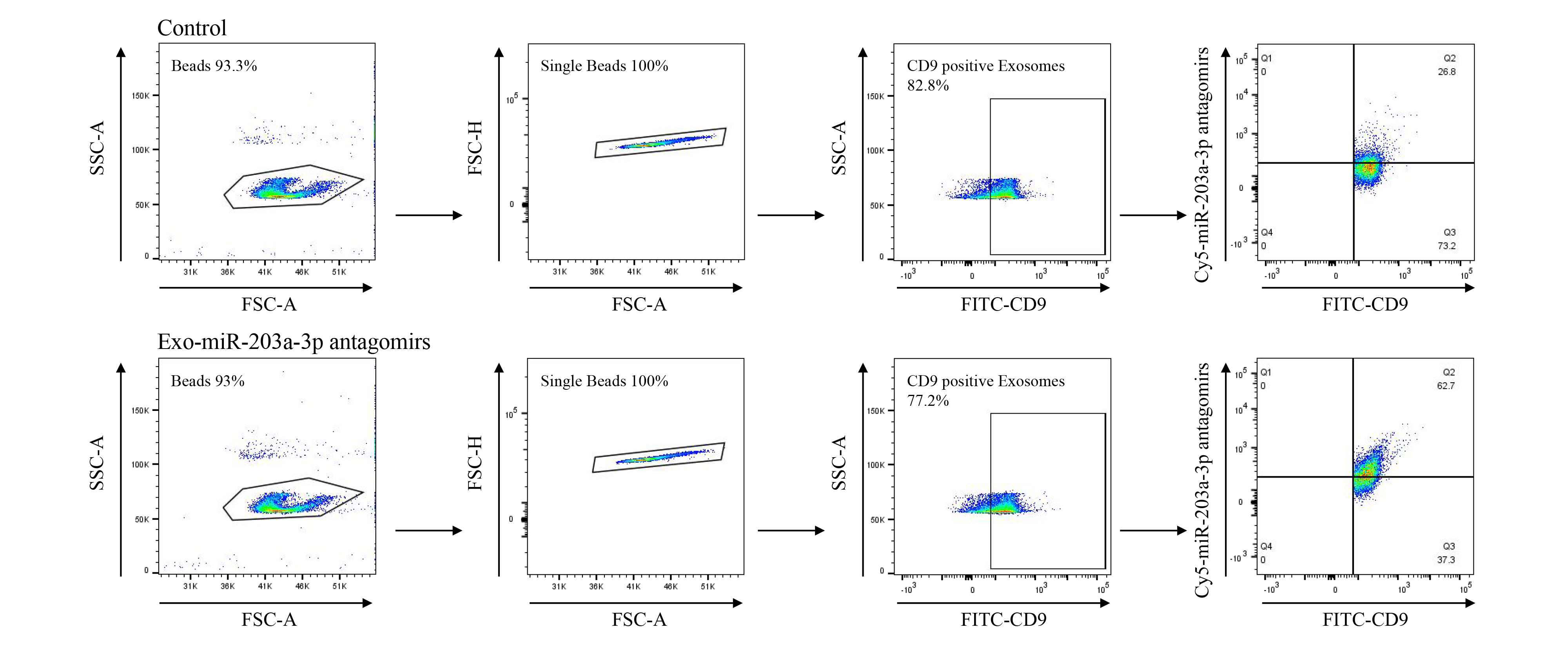


**Fig. S8 The encapsulation efficiency of exosomes loaded with Cy5-miR-203a-3p antagomirs**

Cy5-miR-203a-3p antagomirs in exosomes derived from RWPE-1 cells were detected by flow cytometry. The control was exosomes mixed with miR-203a-3p antagomirs without electroporation.

**Supplementary Table 1: Data of sequences for cell transfection in this study**

| Name | Sequence (5' to 3') | |
| --- | --- | --- |
| miR-203a-3p mimics NC | sense | UUCUCCGAACGUGUCACGUTT |
|  | antisense | ACGUGACACGUUCGGAGAATT |
| miR-203a-3p mimics | sense | GUGAAAUGUUUAGGACCACUAG |
|  | antisense | AGUGGUCCUAAACAUUUCACUU |
| miR-203a-3p inhibitors NC | sense | CAGUACUUUUGUGUAGUACAA |
| miR-203a-3p inhibitors | sense | CUAGUGGUCCUAAACAUUUCAC |
| miR-203a-3p antagomirs NC | sense | CAGUACUUUUGUGUAGUACAA |
| miR-203a-3p antagomirs | sense | CUAGUGGUCCUAAACAUUUCAC |
| si-NC | sense | UUCUCCGAACGUGUCACGUTT |
|  | antisense | ACGUGACACGUUCGGAGAATT |
| si-DUSP5-1 | sense | GAGACUUUCUACUCGGAAUTT |
|  | antisense | AUUCCGAGUAGAAAGUCUCTT |
| si-DUSP5-2 | sense | GCAUGGCUUACCUUAUGAATT |
|  | antisense | UUCAUAAGGUAAGCCAUGCTT |
| si-DUSP5-3 | sense | CACCUACACUACAAAUGGATT |
|  | antisense | UCCAUUUGUAGUGUAGGUGTT |

**Supplementary Table 2: Data of sequences for qPCR in this study**

| Gene | Sequence (5' to 3') | |
| --- | --- | --- |
| Human |  |  |
| GAPDH | Forward: GTGGAGTCCACTGGCGTCTT | Reverse: GTGCAGGAGGCATTGCTGAT |
| MCP-1 | Forward: AGCAGCAAGTGTCCCAAAGA | Reverse: TTGGGTTTGCTTGTCCAGGT |
| IL-6 | Forward: CAGTTCCTGCAGAAAAAGGCAA | Reverse: AGCTGCGCAGAATGAGATGA |
| IL7 | Forward: TTTTCCTGCGGTGATTCGGA | Reverse: CTACTTTGCGCTTGGTCTGC |
| IL15 | Forward: AGTTGGCCCAAAGCACCTAA | Reverse: GCACTGAAACAGCTGCACAA |
| IL31 | Forward: TGCATGGACCTCGCACTAAA | Reverse: GAATCACGGCAGAGTTCCCA |
| CXCL1 | Forward: CTGGCTTAGAACAAAGGGGCT | Reverse: TAAAGGTAGCCCTTGTTTCCCC |
| CCL3 | Forward: CGAGCCCACATTCCGTCAC | Reverse: GATGCAGAGAACTGGTTGCAG |
| CCL11 | Forward: CCCAGCCAGCTGTGGTATTC | Reverse: CGGGCTCAGGTGACCTTC |
| IL-17A | Forward: CACCTTGGAATCTCCACCGC | Reverse: GGATCTCTTGCTGGATGGGG |
| CXCL12 | Forward: TGCCCTTCAGATTGTAGCCC | Reverse: CGAGTGGGTCTAGCGGAAAG |
| Rat |  |  |
| GAPDH | Forward: CTCTCTGCTCCTCCCTGTTC | Reverse: CGATACGGCCAAATCCGTTC |
| MCP-1 | Forward: AGCCAACTCTCACTGAAGCC | Reverse: GGGGCATTAACTGCATCTGG |
| Mouse |  |  |
| GAPDH | Forward: GCCTCCTCCAATTCAACCCT | Reverse: CCCAATACGGCCAAATCCGT |
| MCP-1 | Forward: TGACCCCAAGAAGGAATGGG | Reverse: ACCTTAGGGCAGATGCAGTT |
| DUSP5 | Forward: TGCACCACCCACCTACACTA | Reverse: CTTCTTCCCTGACACAGTCAAT |

**Supplementary Table 3: Classification of prostatic inflammatory changes.**

| Feature | Details |
| --- | --- |
| Glandular/peri-glandular inflammatory changes | |
| Grade 1: mild | Individual inflammatory cells, most of which are separated by distinct intervening spaces |
| Grade 2: moderate | Confluent sheets of inflammatory cells with no tissue destruction or lymphoid nodule/follicle formation |
| Grade 3: severe | Confluent sheets of inflammatory cells with tissue destruction or nodule/follicle formation |
| Extent of prostatic inflammatory changes | |
| Grade 1 | focal (<10%) |
| Grade 2 | multifocal (10%-50%) |
| Grade 3 | diffuse (>50%) |

**Supplementary Table 4: The common genes of hsa-miR-203a-3p predicted by four databases**

| 11 common elements in miRBD, TarBase v.8, miRTargetLink, and DIANA | |
| --- | --- |
| SNAI2 | ARPP19 |
| **DUSP5** | PCGF6 |
| ABCE1 | GSK3B |
| DLX5 | CREB1 |
| IL24 | NETO2 |
| NCL |  |

**Supplementary Table 5: microT-CDS predicted interactions for hsa-miR-203a-3p**

| Gene Name | Score |
| --- | --- |
| **DUSP5** | 0.987 |
| CRK | 0.958 |
| MEF2C | 0.947 |
| MAP3K1 | 0.945 |
| RAP1A | 0.935 |
| PRKCB | 0.926 |
| MAPK3K5 | 0.921 |
| PPM1A | 0.914 |
| TAOK1 | 0.897 |
| NTRK2 | 0.814 |
